# Current understanding of epigenetics mechanism as a novel target in reducing cancer stem cells resistance

**DOI:** 10.1186/s13148-021-01107-4

**Published:** 2021-05-29

**Authors:** Saeedeh Keyvani-Ghamsari, Khatereh Khorsandi, Azhar Rasul, Muhammad Khatir Zaman

**Affiliations:** 1 Department of Microbiology, Karaj Branch, Islamic Azad University, Karaj, Iran; 2 Department of Photodynamic, Medical Laser Research Center, Yara Institute, ACECR, Tehran, Iran; 3 Department of Zoology, Government College University Faisalabad, Faisalabad, 38000 Pakistan; 4 Department of Biotechnology, Abdul Wali Khan University Mardan (AWKUM), Mardan, 23200 Pakistan

**Keywords:** Cancer stem cell (CSC), Epigenetic modifications, Signaling pathway, Drug resistance, Epi-drugs

## Abstract

At present, after extensive studies in the field of cancer, cancer stem cells (CSCs) have been proposed as a major factor in tumor initiation, progression, metastasis, and recurrence. CSCs are a subpopulation of bulk tumors, with stem cell-like properties and tumorigenic capabilities, having the abilities of self-renewal and differentiation, thereby being able to generate heterogeneous lineages of cancer cells and lead to resistance toward anti-tumor treatments. Highly resistant to conventional chemo- and radiotherapy, CSCs have heterogeneity and can migrate to different organs and metastasize. Recent studies have demonstrated that the population of CSCs and the progression of cancer are increased by the deregulation of different epigenetic pathways having effects on gene expression patterns and key pathways connected with cell proliferation and survival. Further, epigenetic modifications (DNA methylation, histone modifications, and RNA methylations) have been revealed to be key drivers in the formation and maintenance of CSCs. Hence, identifying CSCs and targeting epigenetic pathways therein can offer new insights into the treatment of cancer. In the present review, recent studies are addressed in terms of the characteristics of CSCs, the resistance thereof, and the factors influencing the development thereof, with an emphasis on different types of epigenetic changes in genes and main signaling pathways involved therein. Finally, targeted therapy for CSCs by epigenetic drugs is referred to, which is a new approach in overcoming resistance and recurrence of cancer.

## Introduction

**Fig. 1:**
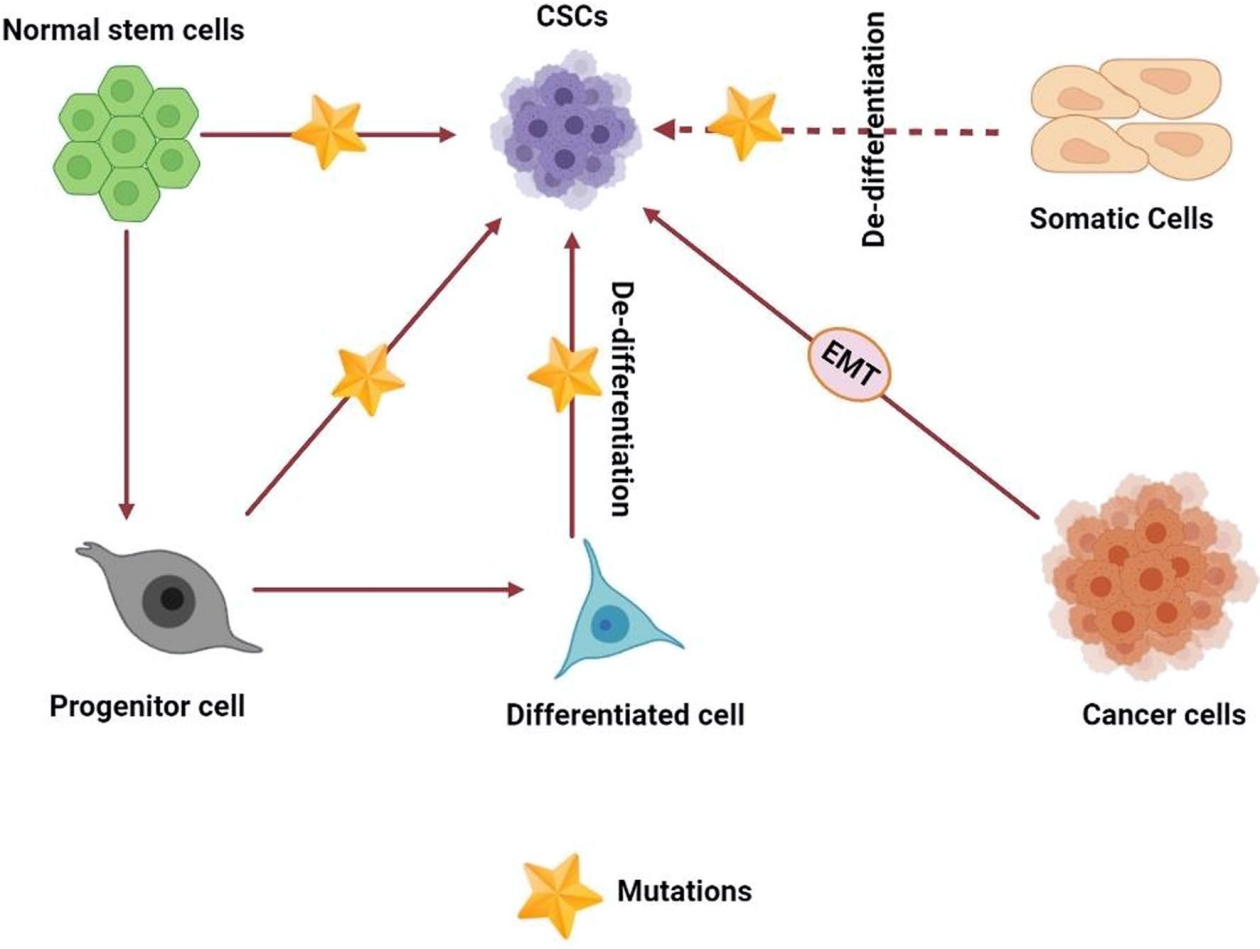
Proposed models for cancer stem cells (CSCs) origin in cancer development. In the normal differentiation process, a cell differentiates to form two cells, differentiated and primitive. A finally differentiated cell is formed from precursor progenitor cell and eventually subject to apoptosis. CSC may originate from a normal stem cell, a normal progenitor cell, or a normal differentiated cell by genetic mutation which will activate self-renewal genes in them. Also cancer cells via EMT can change to CSCs

Despite the myriad of studies and advances in cancer treatment, cancer remains one of the main causes of death globally. The rapid detection of cancer is significantly beneficial in the treatment thereof through common therapy such as surgery, chemotherapy and radiotherapy. However, a small subpopulation of cells have been detected in various tumors that are resistant to therapy and remain inside the tumor. Here, these cells grow and cause recurrence, metastasis and spread of new tumors. Being referred to as cancer stem cells (CSCs) and [1], having the ability of self-renewal and differentiation into different cell types. Due to these characteristics, CSCs can form heterogeneous lineages of cancer cells which are also preserved during metastasis. CSCs heterogeneity is one of the main problems of treatment because these cells ensure the survival of cancer cells in difficult conditions and are resistant to treatment [2–4]. Therefore heterogeneity of CSCs increases the viability of tumor cells and their invasion into other tissues [5, 6]. CSCs are located in an microenvironment called niche, which help them to maintain their characteristics [7]. Different theories have suggested that CSCs may have been produced from normal stem cells/progenitor by oncogenic mutations or from cancer cells and or differentiated cells by abnormal genetic and epigenetic changes (Fig. 1) [8]. CSCs have specific markers that distinguish them from other tumor cells, several of which are specific to different types of cancer and even different stages of cancer development [5]. CSCs have a high ability to metastasize, which can lead to recurrence of the disease years after primary tumor treatment [9]. A plethora of evidence has demonstrated that genetic and epigenetic changes by inducing self-renewal, differentiation and metastasis in CSCs, render significant resistance to chemo- and radiotherapy [1, 10–13]. CSCs can spread the cancer in sites farther from the main tumor, survive and multiply in the new organs, and thus cause metastatic tumors, which leads to the return of the disease and is known as one of the main causes of death in patients [14, 15]. Moreover, studies have shown that CSCs can exit the cell cycle and enter a state of quiescence (G_0_ phase) that increases the survival thereof against treatment. Therefore, part of the resistance of CSCs can also be attributed to the quiescence state and lack of DNA synthesis, while most chemotherapeutic agents affect cells that actively divide and synthesize DNA [10]. In addition, with regard to excision repair cross-complementation group 1 (ERCC1) expression involved in DNA nucleotide excision repair (NER) pathway and O(6)-methylguanine-DNA methyltransferase, observations have been made that a DNA-repairing enzyme is higher in CSCs than non-CSCs [7, 16]. Researchers have also elucidated that, compared with other tumor cells. DNA damage checkpoint responses such as NBS1, Chk1 and Chk2 are more expressed in CSCs, which can help repair damaged DNA caused by drugs. Overexpression of DNA-repairing kinases in CSCs also protects DNA from radiation-induced damage, with the DNA damage repair system in CSCs actually being more efficient [10]. In addition, overexpression and high activity of ROS scavenger enzyme such as catalase, superoxide reductase, superoxide dismutase, glutathione reductase, glutathione peroxidase has been observed in CSCs that reduces the ROS produced during treatment compared to other cancer cells. Reducing the ROS generation reduces the damage caused by chemotherapy and radiotherapy to the DNA, lipids, and cell proteins in CSCs [15, 17, 18]. Overexpression of multi-drug resistance (MDR) proteins such as ATP-binding cassette (ABC) transporters in CSCs is another factor in the resistance mechanism of them. For example, expression of ABCB1, ABCB5, ABCC1, and ABCG2 in CSCs has been reported in association with the resistance of these cells to chemotherapy [15, 19].

Abnormal epigenetic alternations in DNA methylation, histone modifications, RNA methylations, and noncoding RNAs have been demonstrated to be vital in maintenance and survival of CSCs, tumor initiation and progression [11, 20, 21]. It was observed that hypermethylation of the promoters and deregulation of histone modifications, inhibited the expression of tumor suppressor genes in the CSCs [22, 23]. Key signaling pathways which regulate self-renewal of adult stem cells, including Wnt/β-catenin, Notch, Hedgehog, and TGF-β/BMP, are altered in CSCs through epigenetic changes. Theses deregulation can maintain the stemness, differentiation, and resistance of CSCs to drugs [10, 12].

As widely regarded, CSCs are resistant to conventional treatment and have been identified as one of the important challenges to overcome in cancer therapy [24]. Recently, CSCs have been considered for the design of new antitumor drugs that target epigenetic changes and pathways involved in the formation thereof [6, 25]. The most important epi-drugs are inhibitors of DNA methyltransferase and histone deacetylase, which have exhibited favorable results in the elimination of CSCs and cancer treatment [26].

For the aforementioned reasons, identifying the characteristics of CSCs and the factors affecting their generation and maintenance thereof is important for targeted cancer treatment. In the present review, the existing research is explored on CSC features, epigenetic modifications, and the impact thence on key signaling pathways in CSCs resistances. Finally, it promises new insights into epigenetic targets for CSC resistance eradication are revealed.

## Cancer stem cells: biology, origin, and their niche

Cancer stem cells were identified in the late 1990s and have been of considerable interest to scientists throughout the twenty-first century. In acute myeloid leukemia (AML), Bonnet and Dick first detected cells having similar characteristics to normal hematopoietic stem cells with the same markers (CD34^+^ and CD38^−^) that were much more prone to developing leukemia, which were referred as leukemic stem cells (LSCs) or CSCs [6]. CSCs were subsequently found in solid tumors such as breast, brain, colon, glioblastoma, pancreas, lung, prostate, and melanoma [25], accounting for about 0.1–10% of tumor cells [27]. CSCs have been identified as tumor initiator cells that also have an integral function in development, metastasis, recurrence, and resistance to treatment [28].

The presence of markers on the surface of CSCs, such as receptors and antigens is one of the best diagnostic methods for these cells in different tumors. Yet, the expression of these markers in CSCs is lower than tumor cell-specific antigens (tumor markers). Furthermore, definition of "marker" is not linked to its overexpression, but their characteristic feature is that they are expressed in a specific cell class similar to stem cells [27]. These markers can be located within the cytoplasm or located on the cell surface and act as surface antigens [5]. By illustration, the high activity of aldehyde dehydrogenase1 (ALDH1) in the cytoplasm is known as a marker of CSCs in a range of cancers, such as breast, endometrial, gastric, leukemia and colon cancer [5, 27, 29]. Numerous studies have discovered a wide range of specific markers related to CSCs. In general, as major markers of CSCs, the expression of CD133, CD24 and CD44 has been identified in solid tumors, while CD34 and CD38 are the most important markers in blood cancers [29]. Notably, the expression level of these markers and their isotypes thereof are diverse in different types of tumors [27]. As an example, ESA+/CD44+/CD24−, and ALDH1+ were detected in breast cancer stem cells (BCSCs); CD133+ in colon, glioblasoma, gastric and lung; EpCAM, CD133/EpCAM, CD90+ ESA+CD133+ CD44+CD24+ in liver; and ESA+/CD44+/CD24+ in pancreatic CSCs (Table 1) [25, 30–32].

**Table 1:** Example of some surface marker of CSC in different human cancers [27, 33–35]

Cancer	Gender	Marker	Stemness-associated markers	References
Head and neck	HNC is more common in men by twofold–fivefold compared to women	CD44, CD271	The highest levels of CD44 were observed in patients with advanced stages of disease as compared to a healthy control group Using both CD44 and CD271 allowed the isolation of CSCs from HNSCC	[36–41]
Breast	Breast cancer is so common in women and—less than 1% of breast cancers occur in men	ALDH1, CD44, CD133, CD24	ALDH1^+^CD44^+^/CD24^−/low^ cells demonstrated the strongest stem-like properties ALDH1 marker is a good predictive marker for breast cancer CD133 expression was decreased in tumors with larger tumor size, higher stage and lymphovascular invasion CD133 expression was correlated with positive HER2 status	[42–46]
Prostate cancer stem cell	Not found	CD44, CD 133	CD133^+^ cells were demonstrated to be able to possess a high in vitro proliferative potential	[47]
Ovarian	Not found	CD133, CD44, CD117, CD24	CD44 is one the potential marker of ovarian cancer CD 133 is one of the most commonly reported ovarian CSC surface markers CD24 is associated with tumor formation, metastasis, poor prognosis, chemoresistance, and recurrence of disease	[48, 49]
Colon	Higher colon cancer age-adjusted incidence among men than women	EpCAM, CD44, CD29, CD24, CD133, CD166	CD133 is considered a specific marker of primary colorectal CSCs CD166 can be considered together with other markers, such as CD44, CD24, CD29 and CD26	[50–53]
Renal cancer stem cells	Renal cell carcinoma occurrence 2 to 3 times higher in men than in women	CD105, ALDH1, OCT4, CD133	CD44−CD105− displaying stem-like phenotype	[54–56]
Hematological and leukemic stem cells	Females were slightly more affected compared with males	CD19, CD34, CD26, CD38, CD33+	Lack of CD34 or high CD38 expression is associated with favorable prognosis	[57–59]
Bone marrow (BM)	Gender differences was not statistically significant	CD10, CD19 and CD34	BM case is CD19^+^ CD10^+^ B cell precursors and the percentage of CD34^+^ was also higher than normal case. Commonly CD10+ is detected, too	[60, 61]
Brain	Glioblastoma incidence is 60 percent higher in males than in females	CD15, CD90, CD133	CD15 exhibited stable expression in long-term cultured tumor spheres, whereas CD133 expression decreased significantly in late passages CD15 can be used as a marker of stem-like cells derived from brain tumors in all stages	[62, 63]
Hepatocellular carcinoma (HCC)	Hepatocellular carcinoma was more aggressive in male	EpCAM, CD133, CD44, CD90, CD133	EpCAM or CD133 has been used commonly as the tumor initiating cells marker in hepatocellular carcinoma CD90 may be considered to be a marker for invasion, migration, and metastasis	[64–66]
Melanoma stem cells	The majority of people who develop melanoma are white men over age 55	CD271, CD20,	CD271 associated with metastasis and maintains long-term tumor growth	[67, 67]
Endometrial	Endometrial cancer is the sixth most commonly occurring cancer in women	ALDH1, CD133	ALDH cells demonstrated greater endometrial cancer stem cell activity than CD133 cells and had increased expression of stem cell and epithelial–mesenchymal transition gene	[69]
Lung	Men develop lung cancer more often than women	CD44, CD166, CD133	Increased expression of CD44 was significantly correlated with higher grade tumors The expression of the adhesion molecule CD166 in primary lung cancer is associated with smaller tumors with no lymph node metastasis CD166 population shows higher in vivo tumor initiating capacity in comparison to CD133^+^, CD44^+^, and EpCAM^+^ cells isolated from the same cells	[70–72]

Regardless of extensive findings, the origin of CSCs has not been properly identified, although various theories have been presented. Up to date, one approach demonstrates that CSCs can arise from somatic stem cells (Fig. 1) [73, 74]. In addition, deregulation of signaling pathways in adult stem cells, containing Wnt, Sonic Hedgehog ligand, Notch and BMI1 causes the formation of CSCs [73, 75]. Another theory is that progenitor cells are the source of the CSCs. The abundance of these cells in the population of adult tumors relative to stem cells is the reason for expressing this hypothesis. However, these cells have partial ability to self-renewal [76]. Another group of researchers suggests that CSCs originated from differentiated and mature cells that have acquired characteristics similar to stem cells. In fact, it is proposed that genetic and epigenetic changes in differentiation genes, tumor suppressor genes, or signaling pathways related to pluripotency have led to ability of non-stem cancer cells to self-renew and stemness (Fig. 1) [73, 76].

Researchers have shown the prevalence of epithelial to mesenchymal transition (EMT) in cancer cells and its significant association with CSCs, which play a vital role in resistance to chemotherapy (Fig. 1) [77]. EMT is a complex reprogramming system in which epithelial cells miss their differentiation and polarity properties and become mesenchymal cells, leading to increased motility, migration, invasion, dealing with immune system responses, and resistance to apoptosis [78]. It has been seen in the context of cancer, EMT induces stem cell-like features in tumor cells and stimulates the formation of CSCs [77]. Therefore, CSCs show the EMT phenotype. It has been observed that the expression level of E-cadherin in CSCs is low. Simultaneously, the overexpression of mesenchymal markers in them increases the ability of invasion and metastasis in CSCs [79]. Extensive studies have shown that the start of EMT is induced by PI3K-Akt, transforming growth factor-β (TGF-β), epithelial growth factor (EGF), fibroblast growth factor (FGF), signaling pathways such as MAPK/ERK, hedgehog, Wnt/ β -catenin, Notch and NF-кB, along with cytokines such as Interleukin-6 (IL-6) [80–82]. Upregulation of EMT transcription factors increases the ability to initiate tumors in various cancer cells. For example, overexpression of the transcription factors Snail, Twist, and FoxC2 in human mammary epithelial cells increases EMT initiation in cells, leading to the development of phenotypes similar to CSCs [83]. Furthermore, various studies have proved the epigenetic changes, signals from the tumor microenvironment, and dedifferentiation of non-CSC tumor cells to CSCs promote the EMT and the stemness in the cells through affecting the signaling pathways, [79]. For example, it is said that TGF-β plays a vital role in the induction of EMT in normal and disease conditions [84]. Doherty et al. reported the non-stem cell breast cancer cells exposed to TBF-β-induced mesenchymal/CSC-like characteristics in the cells. Inhibition of TBF-β removed the mesenchymal and CSC phenotype, and the cells returned to epithelial and non-CSC phenotype [85]. Another study showed that Wnt and TGF-β act as autocrine signals in breast epithelial cells, activate the EMT pathway in in primary mammary epithelial cells, and ultimately increase migration and self-renewal [86]. Therefore, compounds that eliminate factors affecting in EMT induction are known to be an effective antitumor strategy in CSC eradication [87].

**Fig. 2:**
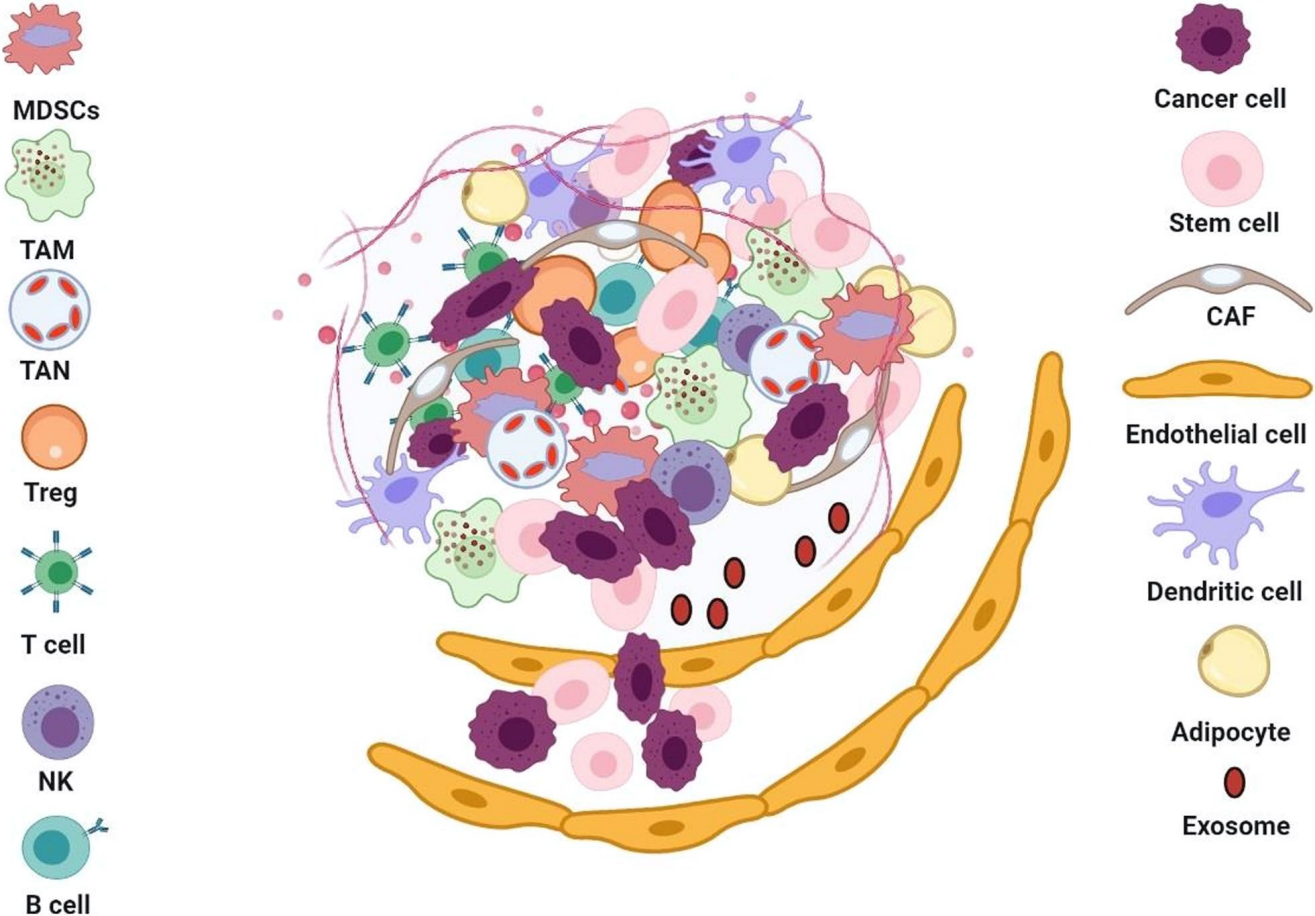
Schematic representation of the cancer stem cell microenvironment or niche. Progression of tumor needs a cooperative interplay between CSCs and their niche. The CSC niche made of various cells including mesenchymal stem cells (MSCs), endothelial cells, cancer-associated fibroblasts (CAFs) and immune cells, tumor-associated macrophages (TAMs), regulatory T cells (Tregs), myeloid-derived suppressor cells (MDSCs), T cells and B cells. These cells secrete various growth factors and cytokines which promote tumorigenesis, tumor progression, and immunosuppression

As it said, CSCs are located in an exceptional area in the tumor microenvironment called the niche. Niche is composed of cell components and secreted factors that preserve the characteristics of the CSCs, maintain their plasticity, protect them from immune responses, induces EMT in them, and increase their ability to invade and metastasize [88, 89]. Fibroblastic cells, immune cells, blood vessels, growth factors, cytokines, and extracellular matrix proteins are present in the niche could affect the CSCs properties (Fig. 2) [89]. As the tumor progresses to more aggressive states, the tumor microenvironment and especially CSC niches determine the fate of CSCs [90]. Also, stromal support cells and other cells in the niche directly and indirectly regulate the number of stem cells, their proliferation, and self-renewal (Fig. 2). In addition to their tumor initiation and progression function, niches protect CSCs from chemotherapy drugs [91]. One of the main components in tumor niche is cancer-associated fibroblasts (CAFs) engaged in the induction of EMT, angiogenesis, metastasis, and drug resistance. CSCs activate the conversion of normal fibroblasts to overproliferative CAFs by the upregulation of Wnt signaling pathway and activating Hedgehog signaling in niche [73]. Another study states that a group of CAFs with CD10 and GPR77 markers produces niches for CSCs that help CSCs survive and promote tumor progression and resistance to chemotherapy. Other studies also have suggested that one of the leading roles of CAFs is to produce niches to preserve CSC. CSCs produce CAFs through signaling pathways, and in contrast, CAFs maintain the spread and survival of CSCs by producing niches [92, 93]. CAFs caused drug resistance in cancer by maintaining stemness in CSCs through secreting NRG1 and activating NF-κB, Wnt, and Notch3 signaling pathways [7].

Mesenchymal stem cells (MSCs) are another family of essential cells in that can differentiate into different cell types. They are multipotent and involved in cancer cell proliferation, metastasis, and angiogenesis. MSCs preserve CSCs phenotype by secreting different types of cytokines into the tumor milieu [89]. For example, MSCs increased the population of CSCs by activating the NF-κB signaling pathway through the secretion of IL-6, IL-8, CXCL7, and CXCL12 or BMP2 signaling in various cancers. In addition, mesenchymal stem cells increase the resistance of CSCs to treatment [7, 94]. Tsai et al. reported that MSCs increased the number of colorectal CSCs in mice by activating the IL-6/STAT3 pathway [95]. Another study showed MSCs overexpressed miR-199a in breast cancer that led to abnormal expression of different types of microRNAs and the formation of CSCs [96].

Due to the proliferation of tumor cell leads to hypoxia in the tumor microenvironment, CSCs in niche are mainly associated with microvascular endothelial cells that have a significant role in maintaining CSCs niche in hypoxia conditions. Studies have shown that hypoxia is an essential element of the niche environment that enhance CSC number and maintains stemness [97, 98]. Exposure to hypoxic conditions induces hypoxia-inducible transcription factors (HIFs) in many solid tumors. HIFs have an essential role in regulating the stem cell phenotype through Oct4, SOX2, NANOG markers, MAPK/ERK, Wnt and Notch signaling pathways [99]. Zhang et al. reported HIF-1α-induced stemness phenotype and self-renewal ability in glioblastoma CSCs and inhibited their differentiation, so that its knockdown decreased the tumor’s progression[100]. Another study reported HIF-1α-activated Notch signaling pathways and thereby increased the expression of Notch downstream genes such as *Hes1* and *Hes2*, which play a major role in the preserving leukemia stem cells [101]. Therefore, HIF-1α plays several roles in the tumor, including regulation of angiogenesis in hypoxia, establishing and preserving the CSCs niche, promoting the stem cell phenotype, and increases the ability of self-renewal, proliferation, and tumorigenesis in CSCs. Markers linked with the stem cell phenotype, such as CD44 and CD133, increases under the influence of hypoxia [98]. CSCs under hypoxia can promote angiogenesis through direct or indirect pathways. They can differentiate into vascular endothelium cells, thereby producing their niche or secreting angiogenesis-inducing factors. Angiogenesis-promoting factor VEGF is expressed more in CSCs than non-CSCs [102, 103].

Therefore, CSCs rely on the cells and factors present in a niche to maintain their function and population. They also themselves secrete factors and signaling molecules to maintain niche structure and function. CSCs and niche act together to provide favorable conditions for the maintenance of CSCs and tumor progression [91].

Key molecular signaling pathways, such as Wnt, Hedgehog, Notch, TGF-β, which are precisely regulated in normal stem cells, are deregulated in CSCs. These pathways’ abnormal activity plays a crucial function in maintaining the characteristics of CSCs such as self-renewal, differentiation, survival, and cell proliferation, which are outlined in the next sections [104].

## Drug resistance mechanisms of CSCs

As described in the previous sections, CSCs as a subpopulation of cancer cells play an important role in drug resistance and cancer recurrence due to their self-renewal properties and differentiation into cancer heterogeneous cells. They can remain as a small subset of tumor tissue after surgery, radiotherapy, and chemotherapy and promote the cancer [24]. Drug resistance is one of the major problems in cancer therapies, leading to treatment failure [105, 106]. Multidrug resistance (MDR) refers to a general phenomenon that resists the broad spectrum of drugs, not only to a specific one [107, 108]. MDR severely succeeds in the effectiveness of the treatment of the different drugs with a similar structure [106, 109]. It has been widely investigated that CSCs have significantly endogenous resistance mechanisms against radiation and chemotherapy compared to non-CSCs [104–108]. For example, according to the Bao et al. study, patients with an irradiated therapy regimen showed glioblastoma cells resistance in the second irradiated recommended that CSCs are not eliminated and then began to self-renew after therapy [110]. The mechanism of glioblastoma CSC's high-resistance to radiotherapy is well defined by Carruthers et al.'s study [111]. They reported fundamental activation of DNA damage reaction due to the generation of a higher level of replicative stress in those cells is the primary response to this phenomenon. Thus, targeting DNA damage checkpoint pathways in CSCs may reveal a promising strategy.

Consequently, chemotherapy and radiotherapy treatment is more effective on most non-CSCs but not CSCs [112]. It seems that drug resistance is closely related to the similarities between CSCs and normal SCs [10]. As SCs play an essential role in keeping the pool of cells in an organism; preserve these SCs is biologically crucial and necessary. Consequently, CSCs, by the aid of different mechanisms, escape from cell senescence or apoptosis [113].

Drug resistance in CSCs has been identified through various mechanisms like (1) increase in anti-apoptotic proteins, e.g., Bcl-2, Bcl-X and c-FLIP [114, 115], (2) overexpression of ABC transporter proteins and detoxifying enzymes [115–117], (3) cell cycle quiescence [118, 119], (4) improved DNA repair capacity [115, 116], (5) enhance the activity of aldehyde dehydrogenase (ALDH) [120], (6) active essential pro-survival signaling molecules like as NOTCH, Wnt/β-catenin, and NF-κB [120–122], (7) improvement in activities of phosphatidylinositol 3-kinase (PI3K)/Akt/mammalian target of rapamycin (mTOR), and (8) maternal embryonic leucine zipper kinase (MELK) [114, 123].

CSCs have been well described by the unusual expression of apoptotic and anti-apoptotic proteins, which participate in survival pathways. Upregulation of transcriptional redox-sensing factor Nrf2 increases CSC’s survival rate via promoting Bcl-2 and Bmi-1 gene transcripts. The oncogene BMI-1 is a key component of polycomb repressive complex 1 [114]. Following the durability of CSCs by the high expression of these anti-apoptotic proteins, different efforts have been made in recent years through the direct inhibition of the Bcl-2 family proteins toward therapeutic interventions [124].

ABCC1, ABCB1, and ABCG2 belong to a massive superfamily of ABC transporters recognized as multidrug efflux pumps with ATPase activity. It is demonstrated that upregulation of ABC transporters by CSCs under chemotherapy regime affords protection of CSCs and promote tumor generation [125]. Frank et al. recognized melanoma cancer stem cells by overexpression of ABCB5 (an ABC family member) in these CSCs. It has been shown that ABCB5 can keep melanoma-initiating cells by a pro-inflammatory cytokine signaling pathway [126]. Overexpression of ABCG2 has also been reported in head and neck, lung, brain, osteosarcoma, melanoma, and prostate CSCs [127]. Some studies reported overexpression of ABC transporters also has been identified in side population (SP) of CSCs cells that indicate a stemness phenotype and can be used as candidates for the identification of CSCs [15, 125, 128]. High ABCB1 expression has been observed in SP cells in a number of cancer cell lines compared to other cells [123]. For example, upregulation of ABCB1 and also ABCG2 were found in SP cells in pancreatic cancer cell line [129]. In fact, ABCG2 is known as a marker of CSCs and plays an important role in drug resistance and ABCB1 has also been found in over 50% of resistant tumors [19, 128].

Recently, it is proved that CSCs resistant to chemotherapy could be because they are quiescent in the G_0_ of the cell cycle. Therefore, chemotherapeutic drugs that affect cell proliferation cannot target them [36, 37]. By understanding the signaling processes and their associated factors that contribute to cell quiescence, appropriate strategies can be used. For example, the dormant phenotype often follows by PI3K-Akt signaling reduction or cell quiescence is remarkably related to mitogenic signals inhibition [130]. Researchers are seeking to find a useful approach to overcome the resistance of CSCs quiescence. Prost et al. showed that agonists of peroxisome proliferator-activated receptor-γ via limiting the expressions of hypoxia-inducible factors 2α (HIF2α) and CITED26 could prevent leukemia CSCs from entering the non-proliferating stage, which is an essential modulator in this process.

Another mechanism that plays a role in radiation resistance is the overexpression of DNA repair proteins that participate in promoting DNA repair in CSCs [116, 131–133]. In comparison with the normal tissues or massive tumors, CSCs are resistant to current treatments by the effect on activation of p53 under radiation-induced DNA damage conditions. Consequently, cell cycle arrest and apoptosis are not working accurately. Over different cycles, it causes the accumulation of DNA mutations [134].

Aldehyde dehydrogenases (ALDHs) are a group of NAD(P)+-dependent enzymes with exogenous and endogenous aldehydes detoxification. High ALDH activity could also be considered a CSCs marker in self-renewal, proliferation, differentiation, and drug resistance. Wnt signaling plays a role in transforming dormant CSCs into active CSCs by increasing cell cycle procedure via β-catenin, enhancing the expression of downstream cyclin D1 and MYC. Additionally, MYC increases the expression of the polycomb repressor complex 1 component Bmi-1 and affects the connection of E2F with cyclin E [128]. Based on various investigations, the Notch pathway’s activation is specifically involved in developing cell survival, self-renewal, metastasis and inhibiting apoptosis in CSCs [135, 136]. Deregulation of Notch signaling, specially Notch1 and Notch4, induce self-renewal and metastasis in breast and HCC stem cells. The critical role of the NF-κB pathway in CSCs involves regulating inflammation, self-renewal, or maintenance and metastasis. CD44+ cells manifest the capacity of self-renewal, metastasis, and maintenance of ovarian CSCs with an improved expression of *RelA*, *RelB*, and *IKKα* and mediating nuclear activation of p50/RelA (p50/p65) dimer [137]. Researches have shown that the mTOR signaling pathway is also associated with CSCs metabolism [138]. By an activated mTOR signaling pathway, low folate (LF) stress reprograms metabolic signals show the potential to induce the metastasis and tumorigenicity of lung cancer stem-like cells. As a result, drugs, vaccines, antibodies, and CAR-T cells that target these pathways can be used to develop novel targeted therapy [128]. MELK (a serine/threonine kinase) is expressed in several cancer stem cells populations [139], which implicated in the survival and drug resistance and tumor recurrence in CSCs. According to Kim et al.'s studies, EZH2 is a target of the MELK/FOXM1 Complex and then increases CSC resistance to radiation [140].

Autophagy is another resistance mechanism of CSCs which plays a major role in the maintenance and survival of various CSCs, such as liver, ovarian, breast, pancreatic, osteosarcoma, and gliobastomabrain cells [141]. For example, Pagotto et al. have shown high autophagy activity in ovarian CSCs compared to non-stem counterpart, which plays a fundamental role in maintaining cells and increasing their resistance to chemotherapy. Inhibition of autophagy by reducing CSCs reduced resistance to chemotherapy [142]. Overexpression of autophagy-related proteins such as ATG3, ATG5, ATG7, and ATG12 in colorectal CSCs also increased resistance to photodynamic therapy (PDT) and decreased PDT-induced apoptosis in the cells. Inhibition of autophagy by pharmacological inhibitors increased the sensitivity of colorectal PROM1/CD133+ CSCs to PDT [143]. Self-renewal features give the constant maintenance of the CSC pool through tumor improvement and initiation. Since most of the genes associated with the regulation are oncogenes like *Smo*, *Shh*, *Gli1*, *Gli2*, and *Ptch1*, the hedgehog pathway represents an essential part of self-renewal. Active mutation in these genes is associated with active hedgehogs, one of the main reasons for many human cancers. Therefore, targeting self-renewal pathways is a promising strategy for eliminating the CSCs [144].

While there is much information about CSC characteristics that can help us to target CSCs efficiently, there are many difficulties to overcome before fully achieving. In the first step, it is essential to find a way to target the only CSCs to avoid destroying normal tissue stem cells and reduce side effects. Secondly, combinational therapies for effective tumor eradication needs to be considered since CSCs may not eliminate. Thus, CSCs have not been introduced in targeted clinical treatment, but they will be a promising future treatment.

## The role of epigenetic modifications in cancer stem cells resistance to chemotherapy and radiotherapy

As described in the previous sections, the presence of CSCs plays an important role in resistance to current treatments [145]. Various studies have shown that epigenetic changes significantly induce the CSC phenotype [146–149]. Epigenetic modifications involve inherited cell phenotypic changes without changes in nucleotide sequence [3]. Substantial epigenetic alternations in CSCs are described in detail below.

### Histone methylation

Histone methylation appears mainly in lysine (K) and arginine (R) residues through the binding of a methyl group to nitrogen atoms in side chains of amino acid or at the N-terminal tails by histone methyltransferases (HMT). Histone methylation is completely conserved during the evolution of animals. Histone lysine methylation can be mono-, di-, or tri-methylation which could activate or inhibit the gene expression [146]. For example, trimethylation at histone H3 lysine 4 (H3K4), H3K36, and H3K79 active transcription and methylation of H3K9, H3K27, and H4K20 repress transcription [147]. Therefore, deregulation of histones methylation because of alternation in gene expression causes various diseases and malignancies. Also, investigations have displayed that histone methylation is different in non-cancer cells and CSCs [148]. Histone methylation has a significant function in regulating the gene expression involved in signaling pathways crucial for maintaining CSCs self-renewal such as Wnt, Notch, and Hg pathways [149]. A study on triple-negative breast cancer stem cells (TNBCSCs) showed that H3K27me3 was abundant in the cells and, conversely, the H3K4me2 level, which has an opposite role H3K27me3, was rarely seen. These abnormal modifications affected key signaling pathways in self-renewal of CSCs, such as Wnt and human gonadotropin-releasing hormone (GNRH), thereby increased survival, tumorigenesis and chemotherapy and radiotherapy resistance in TNBCSCs. Overexpression of DOT1L (the H3K79 methyltransferase), upregulated Rho GTPases and survival proteins in CSCs of HNSCC enhance tumor invasion and chemotherapy resistance. Simultaneously, the inhibition of DOT1L by DOT1L specific small interfering RNAs (siRNAs) reduced the invasion of the tumor and increased chemosensitivity [145]. Enhancer of zeste homolog 2 (EZH2) is a histone methyltransferase and member of the Polycomb-group family. Polycomb-group proteins (PcG) regulate gene expression by epigenetic changes. Overexpression of EZH2 by the induction of trimethylation of H3K27 (H3K27me3) and the regulation of gene expression promoted self-renewal and dedifferentiation in oral cancer cells, ultimately leading to tumor progression and invasion [150]. Momparler and Cote reported DNA methylation and polycomb repressive complex 2 (PRC2), a class of PcG proteins, alone or in combination inhibited differentiation in CSCs. As the expression of differentiation programming genes in CSCs was permanently suppressed by histone methylation and/or DNA methylation, these changes were reversible [151]. Alternative research has suggested that EZH2 inhibits the expression of tumor-suppressor genes by inducing H3K27me3. EZH2 absorbed DNMTs in the promoter area of target genes during cancer progression, resulting in DNA and histones’ methylation. These modifications eventually condensed the chromatin structure and silenced the tumor suppressor genes expression. In fact, PcG target genes prefer to be hyper methylated. Due to EZH2 role in promoting the self-renewal of tumor cells, inhibiting them can prevent the formation of CSCs [152]. For example, it has been proven that downregulation of EZH2 in gemcitabine-resistance pancreatic cancer cells increases the sensitivity of cells to gemcitabin through reducing the number of CSCs [153]. Several EZH2 inhibitors have been specifically synthesized, among which EPZ-6438 (E7438) and 3-deazaneplanocin-A (DZNep) have shown significant anti-tumor activity and have been considered for clinical research [151].

### Histone acetylation and deacetylation

**Fig. 3:**
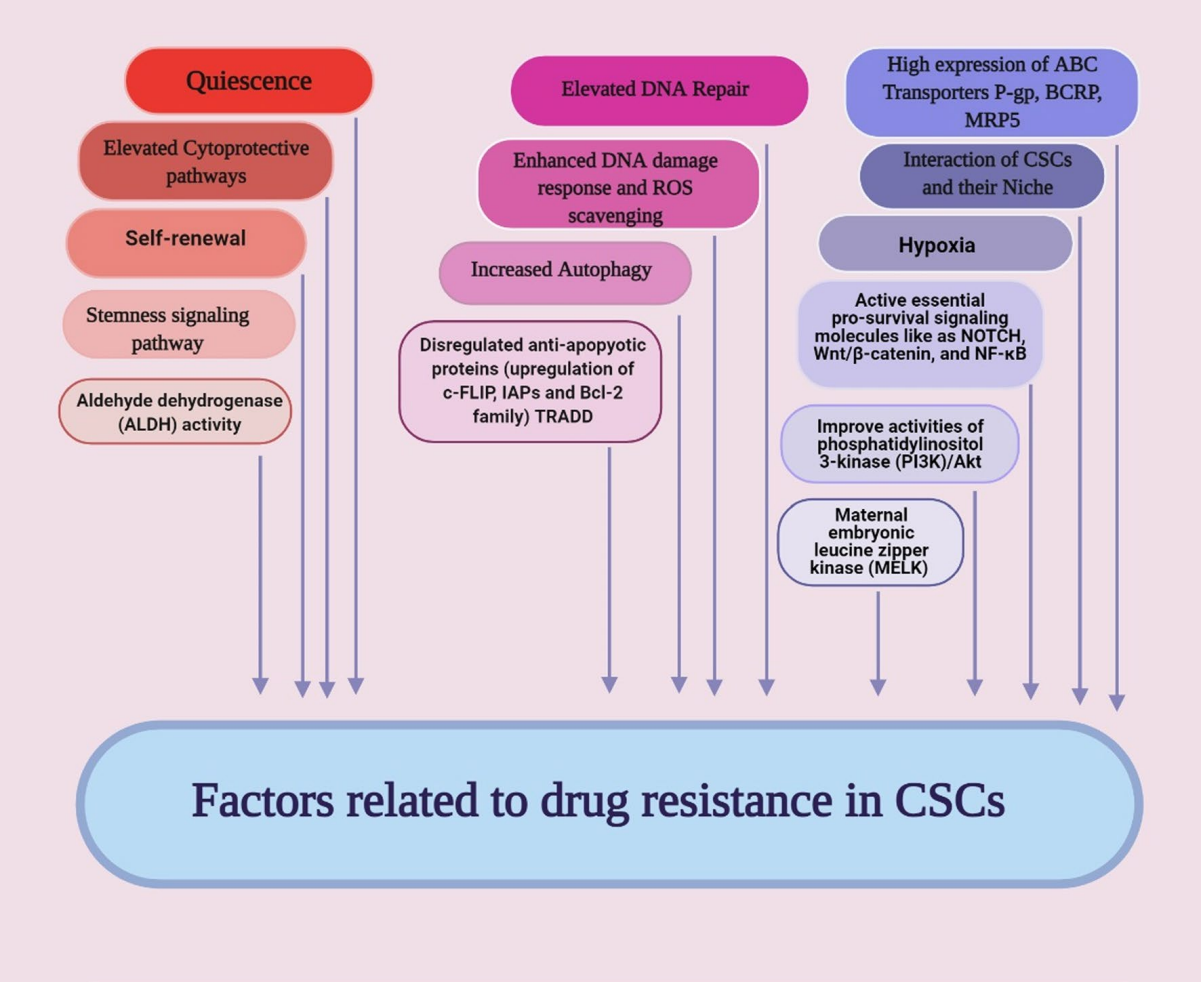
Schematic illustration of factors related to raising resistance in CSCs. Activation of quiescence, cell survival pathways, enhanced drug efflux, the apoptotic signaling disability, enhanced DNA damage repair, enhanced detoxifying activity, and enhanced scavenging of free radicals are feasible agents lead to the CSCs resistance

Acetylation and deacetylation of histones are among the principal epigenetic modifications in various diseases such as cancer [154]. Histone acetyltransferase (HAT) binds an acetyl group to the lysine residue in histone and non-histone proteins, and in contrast histone deacetylases (HDAC) removes the acetyl group from them [155]. HATs are divided into two types, type A and B, based on their location. Type A is placed in the nucleus and type B is located in the cytoplasm [156]. Mammalian HDACs are also divided into four classes, each withseveral members (from HDAC1 to HDAC18) [157]. In general, HATs increase the transcription and expression of genes by relaxing chromatin structure and promoting transcription. HDACs cause the silencing of genes by condensation of the chromatin structure and restricting transcription. The imbalance between acetylation and deacetylation of histones by affecting the signaling pathways of differentiation and self-renewal, leading to the formation and preservation of CSCs [154, 155]. For example, Liu et al. reported HDAC3 and HDAC7 were overexpressed in the CSCs that increased the expression of stem cell markers such as SOX2, Oct4 and Nanog. Inhibition of HDAC3 expression by SiRNA inhibited the proliferation and self-renewal of CSCs. It was also observed the amount of acetylated histones H3 (H3Ac) and H4 (H4Ac) in liver CSCs was lower than non-CSCs and increased histone acetylation led to the cell differentiation [158]. Another study found that HDAC1 and HDAC7, generally enhanced in CSCs compared to non-CSCs and their presence is essential for maintaining CSCs phenotype [159]. HATs also have a vital function in regulating the multiple signaling pathways that maintain cell stemness and differentiation such as Wnt/β-catenin, Notch, Hedgehog, TGFβ/BMP, JAK/Stat, FGF/MAPK and Hippo [160]. For example, CBP/p300 (a member of the type A HATs) has been identified as an essential coactivators of the Wnt/β-catenin pathway in CSCs. As shown, the selectively antagonizing the CBP/β-catenin interaction inhibited drug resistance in CSCs by inducing differentiation in them [161]. CBP and p300 are known as coactivators for hundreds of transcription factors. C-Myb is a transcription factor that is overexpressed in AML cells and is needed for self-renewal, survival, and proliferation of CSCs. Reports indicated that P300 is the main coactivator of c-Myb. Therefore, c-Myb and P300 interaction inhibition can be a promising therapeutic agent in the leukemia treatment [162]. Another study showed the amount of hypoacetylation of histone 3 in HNSCC is higher than normal cells. The deregulation of histone aceylation and deacetylation by affecting the transcription process and gene expression disrupted the processes of proliferation, metastasis, DNA repair, and apoptosis. The HDACs suppression and the induction of the acetylation of histones disrupted the processes essential for the formation of CSCs [163]. Researchers also have shown that HDAC11 overexpresses in CSCs of Non-small cell lung cancer (NSCLC). Inhibition of HDAC11 reduced the expression of Sox2 that is an essential transcription factor for the self-renewal of the cells and their viability. In addition, inhibition of the HDAC11 reduced the lung cancer cells growth and their resistance to treatment (Fig. 3) [164].

### DNA methylation

Alternation in DNA methylation plays a vital function in all stages of cancer, such as the formation of CSCs. This modification is performed by DNA methyltransferases (DNMT1, DNMT3A, and DNMT3B) which transmit a methyl group from S-adenosylmethionine to the position of 5 cytosine bases [12, 165]. The role of DNMT1 is to keep methylation, and DNMT3A, and DNMT3B are accountable for creating de novo methylation [166]. DNA methylation has been seen to be removed or transmitted to future generations without changing the natural structure of the DNA [165]. Methylation is common in areas rich in CPG dinucleotides, titled as CPG islands. CPG islands are abundant in the gene promoter region; methylation of these areas causes long-term silencing of genes [11]. It has been generally reported that hypomethylation in the oncogenes’ promoter increases their expression and hypermethylation in the tumor suppressor genes promoter causes the silencing of genes in many cancers [167]. Decreased expression of tumor suppressor genes has been shown to lead to increased plasticity in cell phenotype such as EMT, change in cell function, self-renewal and differentiation potential, and finally the formation of CSCs in different cancer cells [168, 169]. Although DNA methylation is normally necessary to maintain normal cell function and physiology, its dysregulation leads to tumorigenesis, increased the formation of CSCs and the ability of self-renewal, increased the cell tolerance to drug toxicity, and resistance to chemotherapy [11, 169]. Mutations in epigenetic regulators such as DNMT3A also promote the formation of CSC in tumors. These, mutations that inactivate epigenetic regulatory genes, activate CSC formation pathways and increase resistance in them [170]. For example, among the AML samples which examined, 44% of the genes associated with DNA methylation changes, with the highest mutation observed on DNMT3A [171]. Mutations can occur in stem cells or adult cells, in both cases causing the cell to lose control of its plasticity and original identity [170]. Investigations have shown that DNA methylation regulate the specific CSC genes expression in solid tumors and leukemia. For example, hypermethylation of *WIF1*, *SFRP2*, *SFRP5*, *DKK*, *WNT5a*, *APC* genes, which act as inhibitors of the Wnt pathway, caused these genes to be silenced. Epigenetic silencing of these genes played a vital role in drug resistance in CSCs, and reactivating them induced apoptosis in the cells [172, 173]. DNA methylation regulate the expression of surface markers of CSCs such as CD44 and CD133, as well as ABC transporters such as ABCG2, which is involved in drug efflux from the CSCs and thus promotes chemotherapy resistance [173]. Research has shown that epigenetic changes, such as DNA methylation, cause CSCs to enter a state of cellular quiescence called a dormant state. In this case, the expression of survival and anti-apoptotic genes is increases, which makes the cells resistant to conventional therapies [23]. For example, upregulation of transcription factors FOXG1 and SOX2 caused CSC phenotype in glioblastoma multiforme (GMB). Hypermethylation in downstream targets of FOXG1 and SOX2 including FOXO3 promoted the cellular dormant process in GMB CSCs [174]. Other researchers have shown that breast CSCs can also be dormant by DNA epigenetic changes, although the exact mechanism by which they enter dormant state has not yet been determined. The amount of DNMT1 also increased in mammary tumors and CSCs and aberrant DNA methylation caused resistance to treatment in CSCs by regulating proteins involved in cell growth [175, 176]. Therefore, targeting DNMTs is an effective strategy in regulating tumor suppressor genes and genes related to differentiation, the formation of CSCs, and creating resistance in them.

### RNA methylation

The advancement of technology in recent years, led to the identification the function and regulation of RNA methylation in eukaryotic cells and its importance in biological changes, and the development of related diseases. According to the MODOMICS database, 72 methylation modifications have been identified in RNA, which is about seven times more than those identified in DNA. Most methylation is done on bases in RNA compared to sugar-phosphate backbone [177]. RNA methylation is a post-transcriptional RNA modification that is reversible and affects the RNA stability and translation of mRNA [178]. RNA methylation, especially mRNA and long noncoding RNA, leads to precise regulation of gene expression by impacting the RNA interaction with other cell components [178]. Of all the mRNA modifications, N6-methyladenosine (m6A) is all the more abundant, about 0.1–0.4% of all adenosine. A methyltransferase enzyme complex made up of proteins methyltransferase-like 3 (METTL3), METTL14, and Wilms tumor 1 associated protein (WTAP) which catalyzes this modification [179]. This modification affects all RNA metabolism stages, such as CSC formation, maintenance, and metastasis in various cancers. Deregulation of m6A RNA methylation plays different roles in the cancer initiation, progression and resistance to current treatments [25]. For example, Zhang et al. have shown that hypoxia, which is a definite aspect of the tumor cell environment in breast cancer cells, increase hypoxia-inducible factor (HIF)-1α- and HIF-2α-dependent expression of ALKBH5 as a demethylase that induced m6A demethylation. This led to the demethylation of NANOG mRNA that increased its stability and level. NANOG as a pluripotency factor has an essential function in maintaining the characteristics of the CSCs. Therefore, an increase in NANOG levels led to an increased BCSCphenotype [180]. Cui et al. proposed regulation of m6A RNA modification is significant for the self-renewal of glioblastoma stem cells (GSCs). Downregulation of METTL3 or METTL14 increased self-renewal and tumorigenesis of GSCs. In fact, reducing METTL3 and METTL14 by reducing the m6A RNA methylation levels altered the expression of essential genes in CSCs such as *ADAM19*. While, the use of the inhibitor of m6A demethylase FTO inhibited self-renewal of GSCs and tumor progression [181]. Therefore, CSCs RNA methylation can be considered as a new therapeutic strategy in glioblastoma [182]. Lin et al. have shown that hypoxia reduces METTL3 expression in human sorafenib-resistant HCC. In fact, deregulation of m6A modification of FOXO3 mRNA by downregulation of METTL3 increased the expression of angiogenesis genes, activated autophagy pathways, and stabilized resistance phenotype in the cells [183]. Another study found increased expression of FTO in cervical squamous cell carcinoma (CSCC). FTO reduced m6A methylation in β-catenin mRNA (an EMT maker) and downregulated its expression, which ultimately led to an increase in chemo-radiotherapy resistance in vitro and in vivo [184]. Researches in colorectal cancer (CRC) have also shown that METTL3 upregulates in the cells and through an m^6^A-dependent manner and maintains the expression of SRY-box 2 (SOX2) that is a CSC marker. Therefore, METTL3 acted as an oncogene in CRC that promoted the CRC self-renewal, migration, and tumorigenesis in the cells. The emergence of CSCs in CRC has also led to increased resistance to chemotherapy. So that downregulation of METTL3 in SW620 and HCT116 cells increased the sensitivity of cells to oxaliplatin [185]. In general, m6A modification can affect the onset and progression of cancer through the stability of various mRNA oncogenes, increasing the translation of essential genes for cell survival and regulating the immune system [186]. FTO and ALKBH5 act as an m6A demethylase, their inhibitors such as Rhein, meclofenamic acid (MA) and IOX3 have been identified as effective anti-tumor drugs. However, their clinical studies have not yet been confirmed [186]. For example, Cui et al. proposed that the MA2, the ethyl ester form of MA as a FTO inhibitor increased m6A methylation, thereby inhibiting the growth of GSCs [181]. Also MA also enhanced m6A modification in HeLa cells at a concentration-dependent behavior [187].

### Noncoding RNA as regulator of epigenetic in CSC drug resistance

Noncoding RNAs (ncRNAs) belong to a class of RNAs that do not translate into protein but regulate the genes expression at the post transcriptional level. Therefore, they have been considered as an important epigenetic regulator in recent years. They are distributed into two main families according to their size: small chain noncoding RNAs contain siRNAs, miRNAs, and piRNAs and long noncoding RNAs (lncRNAs) [188]. NcRNAs have vital function in regulating different signaling pathways related to tumor initiation, progression metastasis and resistance to therapies [20]. They are generally aberrantly expressed in various cancer cells and also CSCs. Investigations have revealed that miRNAs (19–24 nucleotide) have an essential function in the biology of CSCs by regulating the signaling pathways of stemness, differentiation, EMT and carcinogenesis in the cells [189]. The abnormal miRNAs expression can work as a tumor suppressors or an oncogene in various cancer cells [190]. For example, miR-21(an oncogenic miRNA) was upregulated in pancreatic CSCs (PCSCs) and through the impact on the PI3K/AKT pathway affected the process of cell proliferation and chemoresistance [191]. On the other side, miRNA-34a (a tumor suppressor miRNA) was downregulated in pancreatic, glioblastoma, and prostate CSCs contributing to the cells’ self-renewal. The restoration of miR-34a in the cells inhibited tumor regeneration and metastasis [192]. The downregulation of miRNA-34a in gastric cancer also affected CSCs formation’s potential by regulating downstream genes including CD44, Bcl-2, Notch, HMGA2, Nanog, Oct4, SOX-2 and YY1. It is shown that miR-34a overexpression suppresses the self-renewal ability of gastric CSCs and can restore the sensitivity of cisplatin-resistant cells [193]. Tsukasa et al. reported the overexpression of miR-30 family including miR-30a, 30b, or 30c upregulated mesenchymal markers such as CD133+ pancreatic CSCs (PCSCs) and increased migratory and invasive capabilities of the cells. Additionally, LncRNAs (ncRNAs over 200 nucleotides in length) have fundamental function in maintaining CSC populations through regulating the expression of stemness transcription factors, their downstream targets, and pathways related to stem cells. For example, downregulation of LncRNA H19 inhibited the expression of CSCs markers such as CD133, Nonag, Oct4, and Sox2 in glioma stem cells (GSCs) [194]. Another study showed that LincRNA-ROR (a large intergenic noncoding RNAs) expression in glioma tumor cells was lower than normal cells. LincRNA-ROR acted as a tumor suppressor gene in glioma cells; its downregulation elevated the self-renewal capacity of GSCs as well as increased the number of CD133+ GSC by the overexpression of stem cell factor such as KLF4 [195]. LincRNA-p21 was also downregulated in colorectal and glioma CSCs. Its overexpression had an anti-EMT activity in the cells and by inhibiting β-catenin signaling reduced the self-renewal of CSCs [196]. Other studies have shown that Lnc34a overexpresses in colorectal CSCs. It recruited DNMT3A and HDAC1 in MIR34A promoter, led to methylation and deacetylation of the promoter. These epigenetic modifications silenced the *MIR34A* gene and thus increased CSCs proliferation [197].

Therefore, due to abnormal expression of ncRNA in CSCs and their role in the carcinogenesis and treatment resistance, they can be considered therapeutic targets. Recently, various clinical trials have been done applying nanotechnology to deliver ncRNAs, that the most studies have been conducted on miRNA [198]. For example, miRNA mimics are used to mimic tumor suppressor miRNAs or anti-miRNAs inhibit the oncogenic miRNAs’ function. Nanoparticles are considered efficient delivery systems for transporting RNAs to protect them from nucleases and deliver them to the target cells with minimal toxicity [199–201]. For example, scientists synthesized a liposomal nanoparticle that delivers miR-34a mimics to the targeted cancer cells and its clinical trial in humans is in phase 1. Also, many clinical trials based on miRNA therapy have been reported in phase 3 or 4 (https://clinicaltrials.gov) [198, 202].

## Epigenetic alterations of signaling pathways in CSC resistance

Several signaling pathways such as Hedgehog, Wnt/B catenin, TGF β/BMP and Notch have a crucial role in maintaining self-renewal, stemness and CSCs differentiation. They are often deregulated in different types of cancer through epigenetic modifications [9]. These abberant epigenetic changes in signaling pathways promote tumor progression, invasion, and resistance by CSCs maintaining [11].

### Wnt/β-catenin signaling pathway

**Fig. 4:**
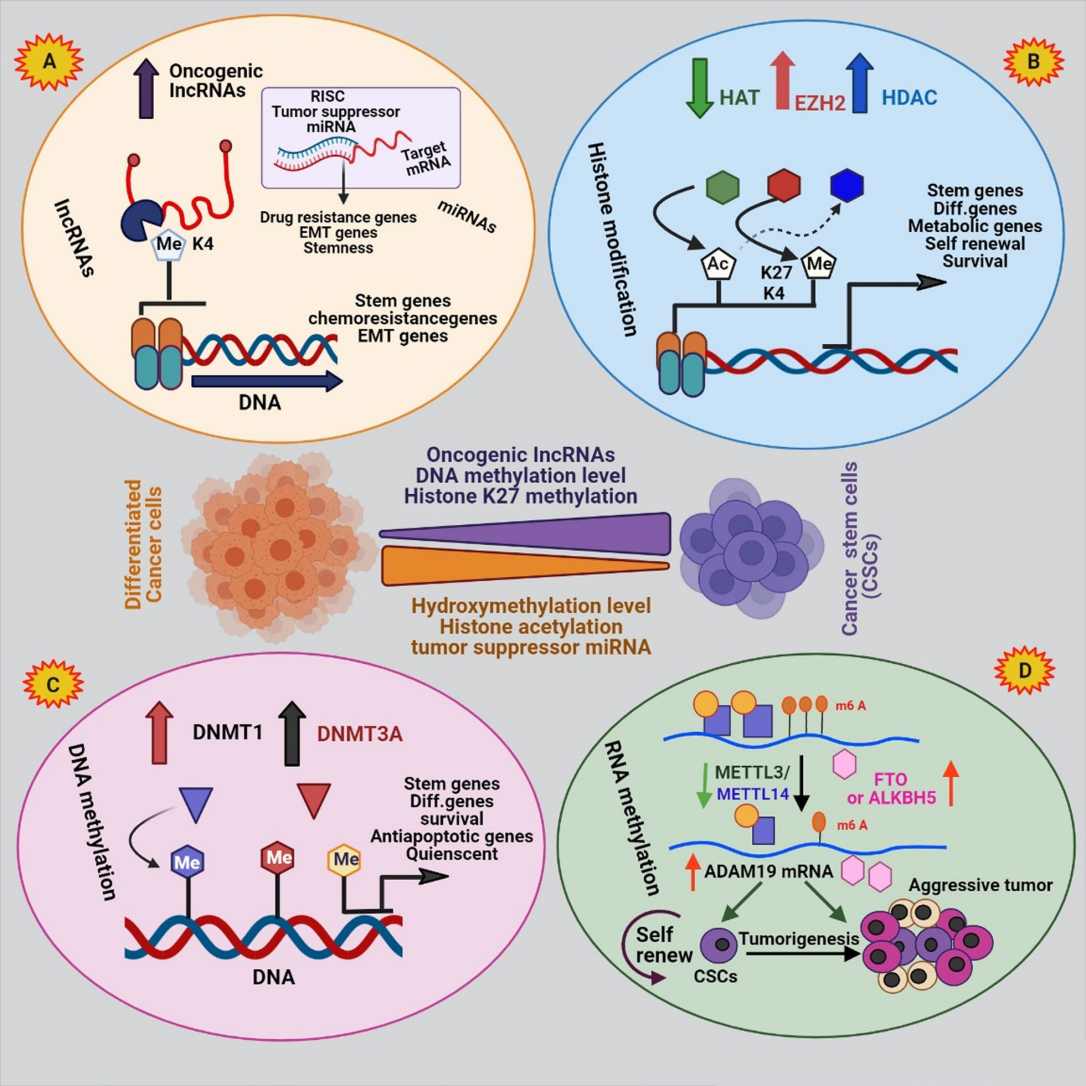
Graphical representation of CSCs epigenetic regulation. Histone modifications, DNA methylation, RNA methylartion and noncoding RNA molecules (lncRNAs and miRNAs) play important role in CSC biology and plasticity. **a** lncRNAs interplay between the various layers of epigenetic gene regulation such as histone modifiers or serving as ceRNAs for miRNAs; while miRNAs can act as anti- or pro-CSC regulators. **b** Histone-modifying enzymes act between CSCs and their non-CSC counterparts, such as HAT, EZH2, and HDAC. **c** The DNMT1 methyltransferase methylates CpG sites relate to methylation of genes vital for stemness feature, differentiation and quienscent of CSCs. **d** RNA methylation increase self-renewal of CSCs

The canonical Wnt/β-catenin signaling pathway modulates gene expression by the transcription factor such as β-catenin, which has a key function in cell proliferation, survival, and maintaining CSCs features. In such a way, high Wnt activity is crucial for initiating and maintaining various tumors [170, 203]. The binding of the Wnt ligands to Frizzled and LRP 5/6 coreceptors at the cell surface stabilizes the cytoplasmic accumulation of β-catenin and transports it into the nucleus. β-catenin acts as an activator of transcription of Wnt target genes such as *c-myc*, *c-jun*, *Axin2*, *EphB/ephrin-B* and *Cyclin D1* in the nucleus, which are crucial for the proliferation and differentiation of stem cells (Fig. 4) [204]. The Wnt pathway also affects the expression of CSCs surface markers such as LGR5/GPR49, CD44, CD24, and Epcam in a variety of tissues [205]. Researches have shown that Wnt/beta-catenin signaling induces CD133 expression in hepatoblastoma cells. Increasing CD133 increased resistance to chemotherapy by maintaining CSCs self-renewal, activating the Akt/PKB and Bcl-2 survival pathways [206]. Overexpression of SOX2 promoted the activation of Wnt/β-catenin pathway in CSCs. The Wnt/β-catenin signaling activated EMT program which led to metastasis and treatment resistance [207]. The Wnt/β-catenin pathway is abnormally activated in various cancers through genetic mutation or epigenetic changes [203]. For example, the researchers showed that promoters of genes contributing to Wnt/β-catenin signaling pathways are hypermethylated in breast cancer cells. Moreover, DNA methylation downregulated the expression of Wnt inhibitors including Wnt inhibitory factor 1 (WIF-1), several frizzled-related proteins (SFRP1-5), and Dickkopf-related protein 1 (DKK1) in the tumor cells [208]. In general, it is revealed that the Wnt/β-catenin signaling activity in BCSCs is more than non-CSCs. Therefore, Wnt/β-catenin signaling activity is higher in BCSCs compared to non-CSCs. Inhibition of the Wnt/β-catenin signaling by reducing the genes involved in CSCs reduced tumor formation and metastasis in the cells [209]. DNA methylation inhibited the Wnt pathway’s gene expression, including SFRP, SOX17 (SRY-box 17), and WIF1 in oral cancer [210]. Modification of histones also affects deregulation of Wnt/β-catenin pathway. The H3K27me3 missing in ASCL1 promoter changed activation of Wnt signaling pathway in GCSCs. ASCL1is a strong regulator of the Wnt signaling pathway that is essential for CSC maintenance and tumorigenicity [211]. H3K4me2 and H3K4me3 also reduced in Wnt/β-catenin pathway in CSCs as compared to non-CSCs which contributed to the progression of CSCs phenotype [22]. It has been reported that the EZH2 is essential for maintaining Wnt/β-catenin pathway in CSCs. Overexpression of EZH2 increased downstream expression on β-catenin genes such as *vimentin* and *c-Myc* in CSCs. EZH2 inhibition by inactivating the Wnt/β-catenin pathway-induced cell arrest in G1/S-phase [212]. Another study reported p300 and CBP, as HAT, are the main coactivators of the Wnt/β-catenin pathway in CSCs, especially β-catenin mediated transcription [161]. MiRNAs have an essential function in regulating of the expression and function of the Wnt-signaling pathway items [204]. For example, the overexpression of miR-19, miR-501-5p, and miR-744 elevated β-catenin activity, thus, enhancing the expression of CSCs proliferative genes, while overexpression of miR-708-5p and miR-142-3p decreased β-catenin activity in CSCs [213]. Another study reported miR-19b, miR-20a, and miR-92a were overexpressed in GCSCs. They played a notable function in self-renewal and maintenance of CSC by targeting E2F1 and HIPK1 which led to the Wnt/β-catenin signaling pathway activation [214]. MiR-148a is a critical tumor suppressor that is reduced in CSCs and its downregulation increase the cell proliferation, invasion, and chemoresistance by acting on the Wnt/β-catenin signaling pathway. The overexpression of miR-148a in cisplatin-resistance colorectal cancer cells inhibited the expression of WNT10b and activity of β-catenin in the cells. Therefore, the expression of markers of CSCs, cell invasion and migration decreased while cell sensitivity to cisplatin was increased [215].

### Hedgehog signaling pathway

**Fig. 5:**
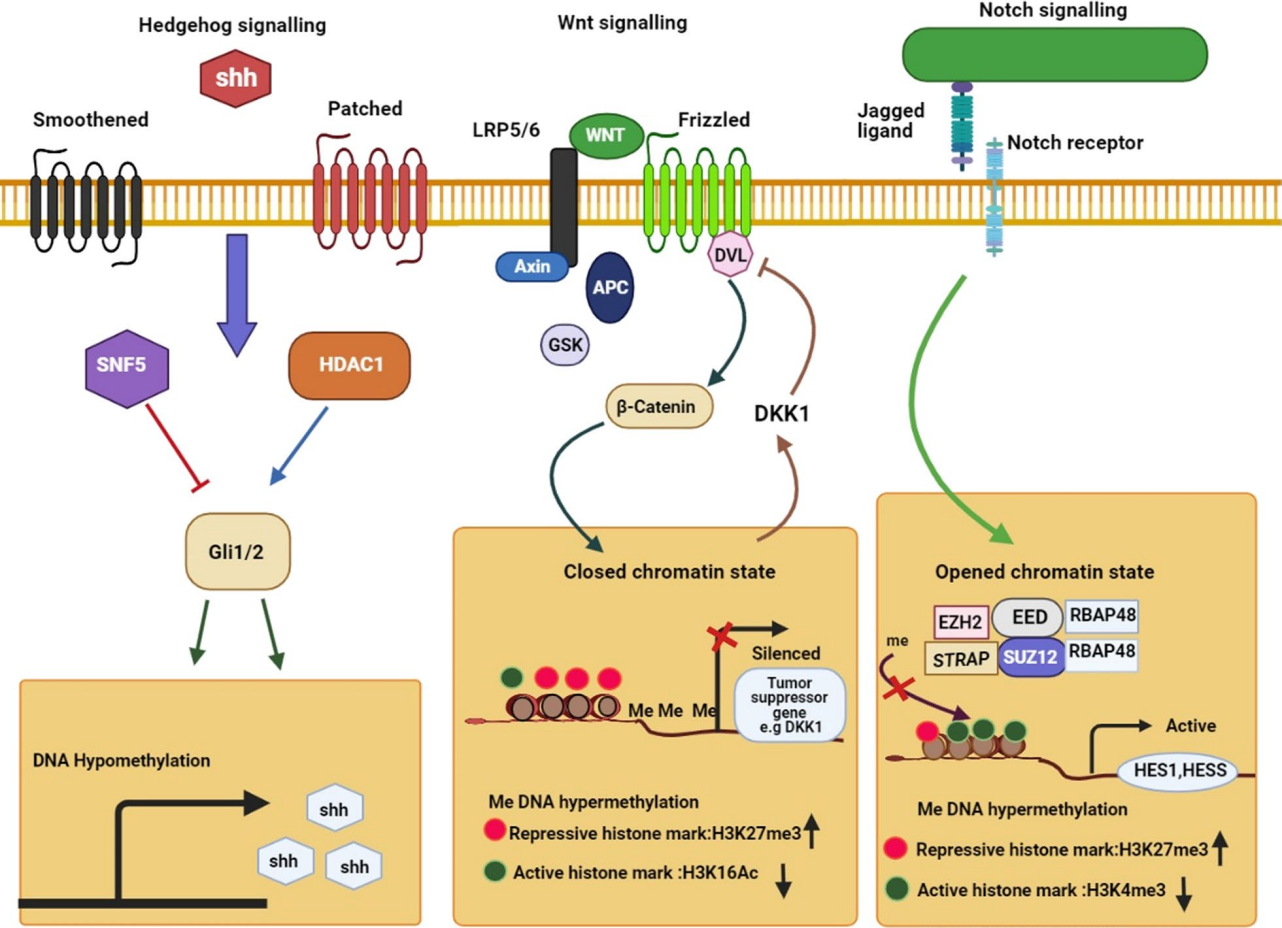
The main CSCs signaling pathways regulation by epigenetic mechanisms. Epigenetic deregulation of CSC-related signaling pathways enables cancer cells to obtain self-renewal properties and drug resistance characteristics. Hedgehog signaling pathway can be activated by Shh promoter hypomethylation and enhance HDAC1 expression. Wnt/β-catenin signaling can be strengthened by reduced DKK1 inhibitor expression of it via promoter hypermethylation and enhanced H3K27me3 and reduced acetylation at H3K16. Notch signaling focus on genes including *Hes1* and *Hes5* which can be active by STRAP at their promoter region

Hedgehog (Hh) is another essential pathway for maintaining self-renewal, the forming CSCs, and chemoresistance. Abnormal Hh signaling pathway activation with different aspects of tumorigenesis causes the development and progression of different types of tumors [216]. Hh signaling also regulates the genes expression of maintaining phenotype and function of CSCs such as Oct4, Sox2 and Bmi1, ALDH1, Wnt2, CCND1, CD44, Twist1, Snail, C-MET, C-MYC and Jagged 1 [128, 217]. Nanog is a key transcription factor for maintaining stemness, self-renewal and differentiation of embryonic stem cell and CSCs, also directly control by Hh signaling pathway [218]. In the presence of Sonic hedgehog ligand (Shh), intermembrane receptor Patched 1 (Ptch1) activates Smoothened (SMO), which in turn causes activation of the Glioma-associated oncogene (Gli) family and their entry into the nucleus. The Gli family controls the expression of Hh signaling target genes as transcription factors (Fig. 5) [217, 219]. Epigenetic modifications can modulate Hh pathway components and lead to the initiation, and progression of various tumors. As an instance, hypomethylation of the Shh promoter region increased the expression of the ligand in breast and gastric cancer cells. Overexpression of Shh activated Hh target genes 101and increased aggressiveness and self-renewal ability of different cancer cells [220, 221]. Histone modifications also modulate Hh signaling pathway. Gli1 and Gli2 proteins are normally acetylated and HDAC1 is essential to activate their transcription. Therefore, overexpression of HDAC1 in cancer cells enhances Hh signaling [219]. Studies showed Gli1 is often active in lung CSCs. The inhibition of Gli1 activity reduced stemness in CSCs and thus decreased tumor growth [222]. Another study reported SMO, Gli and PTCH1 increased tumorigenicity, self-renewal, and migration in CD133+ GSCs. HDAC6 regulated the expression of Gli1, PTCH1, and PTCH2 in these cells [128]. HDAC6 inhibition suppressed Shh/Gli1 signaling pathway in GSCs and increased the sensitivity of cells to radiotherapy [223]. NcRNAs, especially miRNAs and lncRNAs also can regulate Hh signaling activity. For example, Wu et al. reported overexpression of LncHDAC2 increased Hh signaling activity in liver CSCs. LncHDAC2 connected to HDAC2 and reduced the expression of *PTCH1* gene, consequently promoted the self-renewal of liver CSCs and tumorigenesis of hepatocellular carcinoma [224]. MiRNAs are known as the main Hh/Gli signaling pathway regulators. Miele et al. reported re-expression of miR-326 suppressed Hh/Gli signaling pathway by downregulation of Nanog, SMO and Gli2 in medulloblastoma CSCs that impaired self-renewal of cells [225]. Another study found that downregulation of miR-324-5p is associated with increased expression of Hh signaling components such as SMO and Gli1 in myeloma stem cells that increased stemness, survival and drug resistance in the cells [226]. MiR-122 also inhibited lung CSCs self-renewal by targeting the Hedgehog, Notch, and Wnt/β-catenin pathways. Overexpression of MiR-122 increased susceptibility of gemcitabine-resistant NSCLC to chemotherapy and radiotherapy [227]. The researchers demonstrated continuous miR‐302–367 cluster expression inhibited the CXCR4 pathway. The CXCR4 repression inhibited the self-renewal and stem cell-like markers in GCSCs by inhibiting the Shh‐Gli‐Nanog network [228].

### Notch signaling pathway

The Notch signaling pathway is an active pathway in cancer cells that has a key function in cell fate, including proliferation, differentiation, metastasis, angiogenesis, and self-renewal. Aberrant function of this pathway has been seen in various cancers [111]. Owing to the Notch signaling role in maintaining CSCs, inhibition of this pathway can be a useful target for treating cancer by eliminating CSCs. Moreover, due to cross-talk of this pathway with other signaling pathways, including Wnt and Hh pathways, its targeting has been considered in clinical trials [229, 230]. Notch is a transmembrane receptor categorized as, Notch-1, 2, 3, and 4. Activation of receptors by binding to Notch ligands (Delta-like [Dll] 1, 3, 4, and Jagged 1, 2) releases Notch intracellular domain (NICD) into the nucleus and activates target genes [231]. Notch Signaling can promote stemness and EMT in cancer cells by affecting transcription factors connected to EMT and stemness including Snail, Slug, SOX2, Nanog and OCT4 [232]. For example, Notch-1 and JAG ligands overexpressed in colon cancer that increased proteins related to EMT and stemness and promoted CSC phenotype in these cells [233]. Notch signaling can work as an oncogene or tumor suppressor based on cell context [234]. Epigenetic changes affect the Notch pathway items and regulate the activity of this pathway. Researchers have shown the high abundance of H3K4me3 in Notch genes activates transcription of Notch related genes in CRC. conversely, the abundance of H3K27me3 in upstream activators of Notch signaling represses gene expression and decreases the expression of stem cell markers in CRC (Fig. 5) [235]. Wang et al. proved that oxaliplatin enhanced the Notch signaling in CRC by reducing the level of H3K27me3 in the Notch 2 transcription initiation region. The Notch pathway increased the expression of stemness-linked genes in cells and by increasing the CSCs formation leads to greater cell resistance. In contrast, the elevation of H3K27me3 level increased the sensitivity of patients to oxaliplatin [236]. Hyperacetylation in the JAGGED2 promoter overexpresses Notch ligand Jagged2 in myeloma cell lines which has a key function in progression of malignant cells [237]. Another study reported Hairy/Enhancer of Split (HES) as a Notch effector and transcriptional inhibitor, suppressed the Notch pathway by hypermethylation of *DLL1* gene [232]. Improper Notch-Hes1 activity observed in various CSCs, including glioblastoma, breast, pancreatic, and osteosarcoma, plays a central place in stemness, self-renewal, and maintenance of CSCs [238]. High levels of EZH2 had a direct effect on Notch expression and signaling in BCSCs in an independent behavior with the activity of EZH2 histone methyltransferase that led to the development of CSCs populations, and an increase in tumor initiation [239]. MiRNAs also affect the function of Notch signaling in various cancers. For example, a study showed the expression of miR-26a downregulated in osteosarcoma CSCs. MiR-26a overexpression inhibited Jagged1 function and the Jagged1/Notch signaling, thereby reducing the expression of stem cell markers, tumor progression, and enhanced cells’ sensitivity to chemotherapy [240]. MiR-34a also decreased in BCSCs. MiR-34a expression was inversely related to Notch expression and consequently proliferation, migration and invasion of BCSCs. Overexpression of miR-34a enhanced cells’ sensitivity to chemotherapy by reducing the expression of Notch [229]. Ma et al. showed MiR‐129‐5p decreased self-renewal ability, stemness, proliferation, metastasis and chemoresistance in NSCLC by reducing the Notch signaling receptor delta‐like 1 homolog (DLK1) expression [241]. Downregulation of miR424, miR-222, miR-200b, and let-7c in liver CSCs, increased expression of Notch 3, which is one of the essential genes for the activity of CSCs [242]. Downregulation of miR-200 members also promoted Notch pathway activation in pancreatic adenocarcinomas and aggressive basal type of breast cancer by targeting this pathway component, such as Jagged1 and mastermind-like coactivators MAML2 and MAML3. The activation of Notch signalling maintained the CSCS and increased cells survival, stemness and drug resistance [243].

### TGF-β and BMP signaling pathways

Transforming growth factor-beta (TGF-β) is active in different pathways such as cellular homeostasis, cell growth, cell differentiation, and apoptosis in adult and embryonic cells [244]. TGF-β also has an active function in the forming CSCs and resistance chemotherapy and radiotherapy [207]. TGF-β superfamily ligands such as bone morphogenetic proteins (BMPs) bind to type I (that is, signal propagating) and type II (that is, activator) receptors which induce the signaling pathway by activating the SMAD transcription factor in nucleus, open repressive chromatin and regulate target gene expression (Fig. 5) [245]. Scientists have proven that the TGF-β pathway is essential for maintaining the characteristics of CSCs. For example, TGF-β increased the CSC markers’ expression, including CD133 in liver cancer cells, which enhanced the tumor initiation and progression [246]. Kim et al. reported TBF-β-induced EMT in lung cancer cells by downregulation of E-cadherin and overexpression of mesenchymal markers such as vimentin, N-cadherin, slug, fibronectin, and snail. In addition, TBF-β-induced stemness phenotype in the cells by the overexpression of CD87 which could be due to its improper promoter demethylation [82]. Another study reported TGF-β signaling could induce EMT in BCSCs by increasing the population of CSCs to cause treatment resistance. BMP2/7 heterodimer as an TGF-β antagonist decreased TGF-β-driven SMAD signaling and cancer cell progression [7, 247]. TGF-β1 also-induced EMT in hepatocellular carcinoma that increased the density of CSCs [248]. Therefore, deregulation of TGF-β/BMP pathway affects the formation of CSCs [246]. Epigenetic changes can affect the TGF-β ligands or pathway components and TGF-β-related gene expression [245]. Besides, the activated SMAD in the TGF-β/BMP pathway can recruit the epigenetic regulators in nucleus such as HATs, HDACs, DNMTs and lncRNAs which affects the expression of TGF-β target genes [249]. Differentially methylated regions were observed in genes encoding proteins linked to the TGF-β signaling pathway in BCSCs compare to non‐BCSCs. These regions were hypomethylated that led to the overexpression of TGF-β target proteins and affect the regulation of BCSCs differentiation [175]. Another study reported that gene expression associated with the TGF-β signaling pathway increased in chemotherapy-resistant triple-negative breast cancer cells, leading to an increase in CSCs markers. TGF-β type I receptor inhibition and SMAD4 blocked the formation of CSCs and decreased drug resistance in these cells [250]. Scientists have shown that TGFβ1 inhibits DNMT1 and DNMT3β in hepatocellular carcinoma, which led to overexpression of CD133 in the cells by demethylation of CD133 promoter-1. CD133 expression increased the self-renewal, tumor initiation, and resistance to chemotherapy in liver cancer cells [251]. Lee et al. reported that the aberrant methylation of BMP signaling by EZH2 led to the inhibition of the normal differentiation process in glioblastoma stem-like cells and activated their proliferation. Demethylation of the BMP receptor promoter induced the ability of differentiate CSCs and reduced their tumorigenesis [252]. Another study reported miR-495 acted as a tumor suppressor gene in CSCs. Its downregulation induced EMT in the CSCs population in oral squamous cell carcinoma (OSCC) by activating the TGF-β signaling pathway that increased proliferation, migration, and invasion in OSCC [253]. MiR-21 overexpressed about 3–7 times in chemoresistant colon cancer HCT-116 and HT-29 cells rich in CSCs. Mir-21 induced downregulation of TGFβ-receptor-2 (TGFβR2) in the cells. TGF-β demonstrated to affect the Wnt/β-catenin pathway during carcinogenesis. In a way, the TGFbR2 downredulation increased the Wnt/β-catenin signaling and promoted the stemness of colon cancer cells [254]. Pellatt et al. found deregulation of different miRNAs altered the expression of foreign genes in the TGFβ-signaling pathway in colon cancer cells such as TGFBR1, BMP6, BMP2, BMP5, BMP7, BMP7, TGIF1, TGIF2, TFDP1, and TGFβ2 which led to tumor progression [255].

## Epigenetic targeting of CSC

Given the extensive role of epigenetic changes in the development of CSCs and the consequent spread of tumors and their resistance to conventional therapies, targeting them can be an promising strategy in eliminating CSCs and cancer treatment [26, 256]. DNMTis and HDACis as two of the most important epigenetic inhibitors in CSCs are described below. The use of these epigenetic drugs alone, in combination with each other, or with chemotherapy and radiotherapy has provided promising results in the treatment of cancer [256].

### DNMT inhibitors

**Fig. 6:**
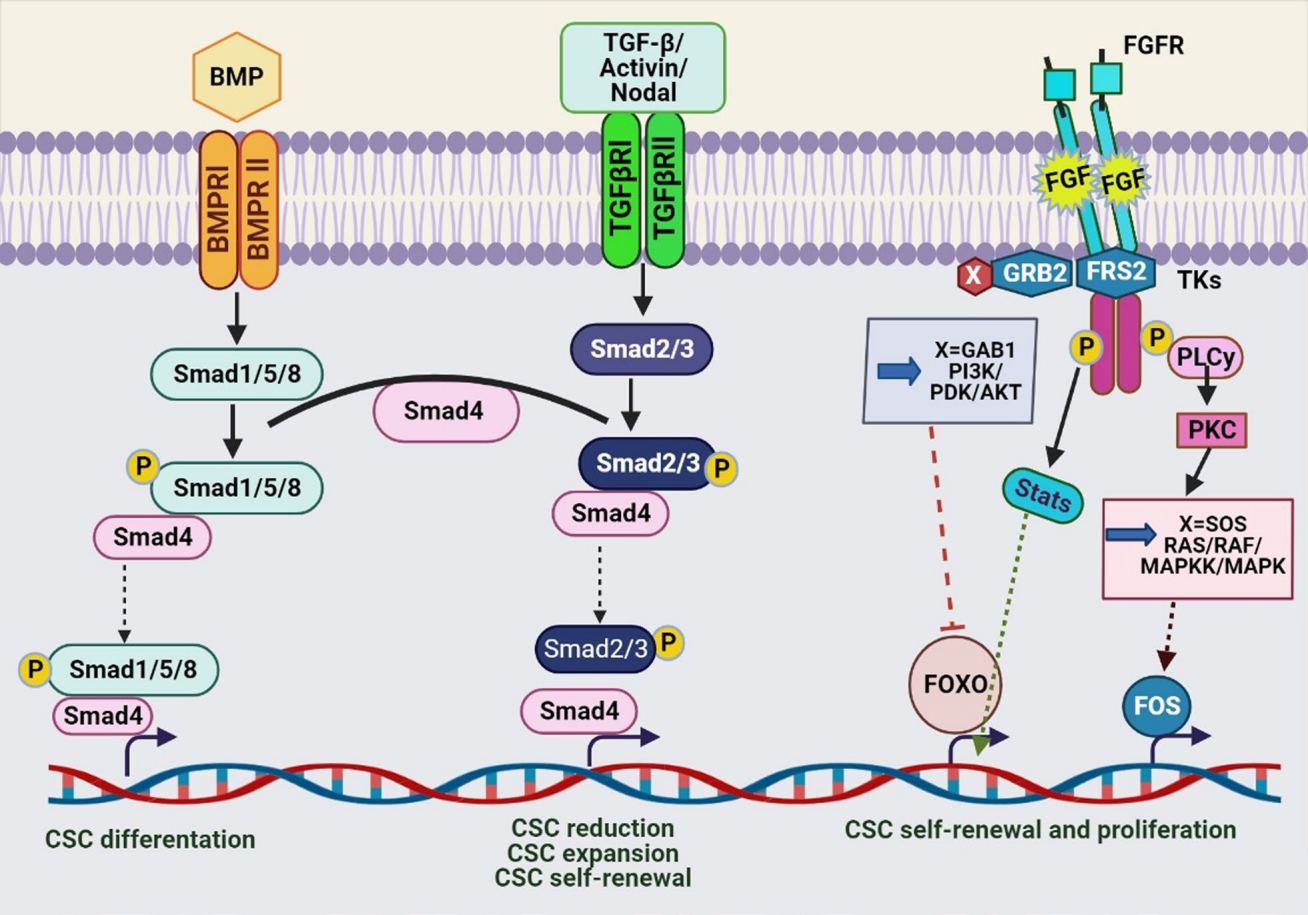
BMP, TGF-β and the FGF pathways in CSCs. BMP signaling is involved in CSC differentiation. The TGF-β/ Activin/Nodal pathway has different function in CSCs according to cancer type such as CSC self-renewal. TGF-β and the FGF pathways have role in pluripotency of CSCs

Studies have shown that DNMTs are essential for the maintaining CSCs, and inhibiting them can reduce tumor development by removing CSCs [257]. DNMT inhibitors (DNMTis) are the first class of drugs prevent epigenetic changes [11]. For example, the researchers showed that inhibition of DNMT1 reduced the lung CSCs proliferation through reducing the methylation of cell cycle regulators and thus inhibited tumor growth [258]. Desitabin and 5-azacitidine (Aza) are the most common DNMTis, which are in the different process of clinical trials in various cancers. They are cytosine analogs that inhibit DNA methyltransferase’s function by participating in DNA structure and covalently binding to enzymes (Fig. 6) [256, 259]. For example, many studies have shown that the use of Aza and 5-Aza-2′-deoxycytidine (AzadC) has successful results in epigenetic therapies. Wongtrakoongate et al. reported AzadC reduced the population of GSCs by upregulation of microRNA-137, which had an inhibitory effect on CSC proliferation. This treatment also decreased the stemness genes expression in prostate and pancreatic CSCs [260]. Decitabine downregulated genes of *OCT4* and *NANOG* in the prostate CSCs. It upregulated the expression of differentiation-related genes, *Nkx3.1*, *CK*5, *CK8*, and *PSA/PSP94*; by inducing differentiation in the cells significantly reduced the self-renewal and tumorigenesis in CSCs [148]. Another study showed that the DNMT1 expression in PCSCs was higher than non-CSCs. Inhibition of DNMT1 with zebularine decreased the tumorigenic and self-renewal capacity of CSCs in vivo and chemoresistance by overexpression and activation of silenced miRNAs during DNA hypermethylation, especially the miR-17–92 cluster. Zebularin is a newer inhibitor that is less toxic and can be taken orally [261]. SGI-110 is a new DNMT inhibitor whose low concentrations on ovarian CSCs led to their reprogramming and reduced tumor initiating ability, and increased cell sensitivity to platinum. In such a way, its use after treatment of ovarian CSC with carboplatin, inhibited the growth of ovarian cells by creating profound hypomethalation in cells in vivo [262]. In general, CSCs are resistant to apoptotic drugs due to specific epigenetic changes in them. For example, Capper et al. showed GSCs are resistant to tumor necrosis factor (TNF)-related apoptosis such as TRAIL-based therapies, and also chemotherapy drugs such as temozolomide, carboplatin, paclitaxel and etoposide due to hypermethylation of caspase 8 promoter [19]. Studies have shown decitabin as a DNMT inhibitor can restore caspase 8 in the cells [263]. Li et al. reported DNMTi 5-Azadc blocked stemness and self-renewal in colorectal CSCs by reducing β-catenin activity and downregulation of the Wnt pathway [257]. Another study showed that DNMT1 inhibition reduced tumor formation and metastasis in human and mouse breast cancer cells by lowering the CSC formation in them. Inhibition of DNMT1 enhanced the expression of the tumor suppressor genes *Isl1* in mammary tumors and CSCs [176]. Overexpression of all DNMTs in gastric cancer led to the downregulation of tumor suppressor genes and critical genes regulating signaling pathways, proliferation, and apoptosis. Low doses of DNMT inhibitors led to silenced genes’ reactivation in gastric cancer and reduced tumorigenic capacity in gastric CSCs [165].

### HDAC inhibitors

**Fig. 7:**
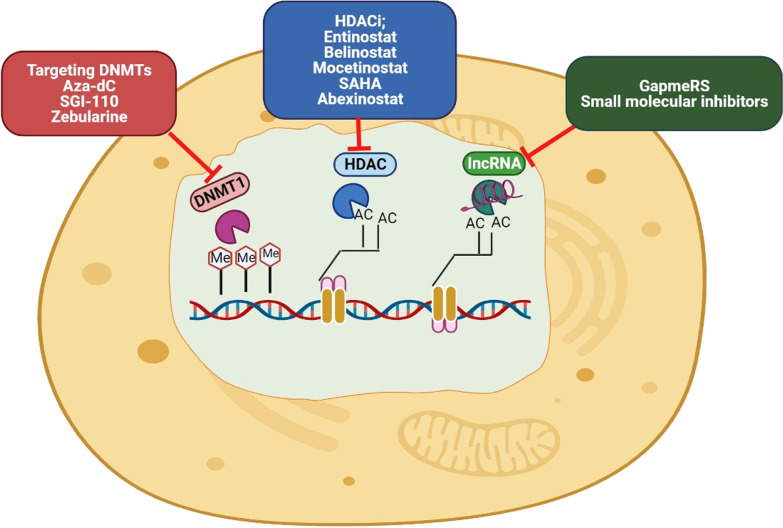
Schematic representation of CSCs epigenome as a target for cancer treatment. Various drugs including Aza-dC (Decitabine), SGI-110 or Zebularine, can reduce DNMT1 protein levels and global methylation. HDAC inhibitors (HDACI), including Mocetinostat, Entinostat and Belinostat, have demonstrated good effect toward inhibitation of HDAC. lncRNAs can be inhibit by GapmeRs or small molecule inhibitors which eventually will be disrupt

As described, deregulation of histone acetylation and the master epigenetic modification in cancer cells, is due to the overexpression and function of HDACs. Their inhibition can be useful in regulating histone acetylation, controlling the population of CSCs [264]. Different types of HDAC inhibitors (HDACis) are classified as pan-HDACis or isoform-selective HDACis. The pan-HDACis target all HDACs, although; most inhibitors have not targeted class IIa HDACs. The selective HDACis inhibit HDACs in a particular class. The selective inhibitors appear to have better clinical outcomes, although this hypothesis has not yet been proven [265]. Many HDACis have been approved by FDA to treat various cancers or are being trialed [256]. These inhibitors, free or in combination with other drugs, inhibit tumor growth by eradicating CSCs. Studies showed inhibition of class I HDACs (HDAC1-3, 8) is very useful in controlling the population of CSCs. In addition to chromatin remodeling, these inhibitors target the stability and activity of essential proteins for the maintenance of CSCs including transcription factors, including Stat3, HIF-1α, Notch1, β-catenin, c-Jun and NF-κB (Fig. 7) [266]. For example, entinostat is a selective inhibitor of class I HDACs that reduced ALDH-1 activity and CSC markers’ expression in triple-negative breast cancer, such as Bmi-1, Nanog, and Oct-4, and by reactivating the E-cadherin gene, it abolished the EMT phenotype. Therefore, entinostat reduced the CSC population and tumor formation in the primary site and metastasis to the lungs [267]. Another study showed that mocetinostat increased the expression of miR-203 in pancreatic cancer. Overexpression of miR-203 reduced the expression of ZEB1 (an activator of EMT) and CSC markers CD24^high^/44^high^ and CD133, suppressed stemness traits and increased cell sensitivity to the gemcitabine [268]. AR-42 (OSU-HDAC42), a pan-HDACi, induced apoptosis in leukemic stem cells by blocking NF-κB and Hsp90 activity but does not effect standard hematopoietic stem and progenitor cells [269]. Suberoylanilide hydroxamic acid (SAHA) is another HDAC inhibitor that significantly reduced the tumor sphere formation and proliferation of head and neck cancer cells by reducing Nanog expression in CSCs. Besides, the SAHA removed cells’ resistance to cisplatin and synergistically enhanced the antitumor effect of cisplatin. Consequently, inhibition of HDACs by reducing CSCs increased the cells’ sensitivity to treatment [270]. Vorinostat (also known as SAHA) is an inhibitor of HDAC-1–3 and HDAC 6. This drug’s clinical trials are being performed alone or in combination with other medicines in the different cancer treatments [256]. Quisinostat, an effective inhibitor of class I and II HDACs, synergized the effect of doxorubicin in breast CSCs and non‑CSCs simultaneously [271]. Salvador et al. reported abexinosta, another pan-HDACi, significantly reduced the population of breast CSCs in cells that expressed low levels of long noncoding RNA Xist and induced differentiation in CSC population from low-dose sensitive breast cancer cell lines [272]. MC1742, and MC2625 are newer pan-HDACis that increased acetyl-H3 and acetyl-tubulin and inhibited sarcoma CSC by inducing apoptosis, cellular arrest and differentiation in them [273].

### Combination of DNMT and HDAC inhibitors

Many studies have reported both DNMTi and HDACi have been approved for the hematologic malignancies treatment by FDA and European Medicines Agency (EMA) [274, 275]. However, in solid tumors, single treatment of these epi-drugs has not yielded favorable results, probably due to their high toxicity, limited bioavailability and low pharmacokinetic effects. However, research has revealed that the combination of HDACs and DNMT inhibitors re-expressed tumor gene suppressor in cancer [275]. High doses of DNMTis cause toxicity, while if combined with HDACis, lower amounts are consumed. Therefore, the combination of these epi-drugs with each other shows potent effects compared to each drug alone and causes synergistic treatment [26]. Aza and decitabine have been approved in the treatment of MDS and AML. They are in Phase II clinical trial in the treatment of ovarian, prostate, and melanoma cancer and combination with HDAC is to treat metastasic melanomas. However, their instability and low bioavailability limited their use [276]. Another study reported the combination of decitabine and SAHA reduced proliferation, self-renewal and EMT processes in PCSCs by reducing expression miR-34a [264]. The phase I/II clinical trials of the combination of Aza and entinostat in patients with refractory metastatic non-small cell lung cancer demonstrated promising treatment results and were utterly tolerable. The general patients’ survival was correlated with these inhibitors’ combined action, which led to the silencing of genes associated with lung cancer [277]. Pathania et al. noted the combination of Aza and butyrate, an HDACi, stopped the growth of CSCs in a mouse mammary tumor model. RNA-seq analysis in CSCs showed that this combination inhibited growth signaling molecules such as RAD51 AP1 and SPC25 by elimination modifications in the chromatin structure [274]. Combination therapy with DNMTi and HDACi also restores the of ER-negative breast cancer cells’ sensitivity to endocrine therapy. These findings support the hypothesis that the combination of DNMT and HDAC inhibitors virtually eliminates CSCs and improves the treatment of refractory and recurrent cancers. However, these inhibitors’ simultaneous use requires a close attention and testing together with clinical barriers that need to be addressed [278] (Table 2).

**Table 2:** Epigenetic drugs in clinical trials

Class	Drug	Treatment method	Cancer type	Current status	Clinical trial	Reference/identifier
DNMTi	Azacitidine	Only	MDS, CMML	Completed	FDA approved	[279]
		Only	AML	Completed	Phase III	NCT01074047
		+ Pembrolizumab	Pancreas cancer	Recruiting	Phase II	NCT03264404
		+ Valproic acid	Advanced cancer	Completed	Phase I	NCT00496444
		+ Quizartinib	Recurrent AML, CMML and MDS	Active, not recruiting	Phase I/ II	NCT01892371
	Decitabine	Only	MDS, CMML	Completed	FDA approved	[280]
		Only	AML	Completed	Phase III	NCT00260832
		+ Fludarabine and busulfan	AML, MDS	Completed	Phase 1	NCT01455506
		+ Quizartinib + venetoclax	Recurrent AML and MDS	Recruiting	Phase I/ II	NCT03661307
	Guadecitabine	Only	AML	Completed	Phase 2	NCT01261312
		Only	HCC	Completed	Phase 2	NCT01752933
		+ Pembrolizumab + mocetinostat	Lung cancer	Recruiting	Phase 1	NCT03220477
	Disulfiram	Only	Metastatic breast cancer	Recruiting	Phase II	NCT03323346
		Only	Prostat cancer	Completed	Not applicable	NCT01118741
HDACi	Romidepsin	Only	CTCL	Completed	FDA approved	[281]
		+ Erlotinib	Stage III/ IV NSCLC	Completed	Phase I	NCT01302808
HDACi	Panobinostat (LBH-589)	+ Bortezomib and dexamethasone	Myeloma who received at least bortezomib and IMiDs	Completed	FDA approved	[282]
		Only	Prostat cancer	Completed	Phase I	NCT00663832
		Only	Colorectal cancer	Completed	Phase II	NCT00690677
	Vorinostat (SAHA)	Only	CTCL	Completed	FDA approved	[283]
		Only	AML	Completed	Phase 2 trial	NCT00305773
		+ Radiation therapy	AML Pancreatic cancer	Terminated	Phase I/II	NCT00831493
		+ Carboplatin + etoposide	SCLC	Terminated	Phase I/II	NCT00702962
		Only	NSCLC	Completed	Phase I	NCT01059552
		Only	Glioblastoma stem cells	Completed	Phase II	[284]
	Chidamide	Only	PTCL	Completed	FDA approved	[285]
		+ Exemestane + placebo	Breast cancer	Active, not recruiting	Phase III	NCT02482753
		+ Paclitaxel + carboplatin + placebo	NSCLC	Completed	Phase II	NCT01836679
	Givinostat (ITF2357)	Only	Lung CSCs	Completed	Phase II	[284]
		Only	Chronic myeloproliferative neoplasms	Active, not recruiting	Phase II	NCT01761968
	Belinostat	Only	PTCL	Completed	FDA approved	[286]
		Only	Advanced cancer	Completed	Phase I	NCT01583777
		+ Ribociclib	Metastatic breast cancer, recurrent ovarian carcinoma	Recruiting	Phase I	NCT04315233
HDMi	Tranylcypromine	+ Tretinoin	AML, MDS, Leukemia	Completed	Phase I	NCT02273102
		+ All-trans retinoic acid + cytarabin	AML, MDS	Recruiting	Phase I/ II	NCT02717884
	Tazemetostat	Only	Malignant mesothelioma	Completed	Phase II	NCT02860286
		Only	Rhabdoid tumors, NI1-negative tumors, synovial sarcoma malignant, rhabdoid tumors of ovary	Recruiting	Phase I	NCT02601937
	CPI-1205	+ Ipilimumab	Advansed solid tumors	Completed	Phase I	NCT03525795
		Only	B cell lymphoma	Completed	Phase I	NCT02395601
ncRNA	EnGeneIC (mir-16 mimic)	Mitoxantrone packaged EDV (EnGeneIC delivery vehicle)	Solid tumors, CNS tumors	Recruiting	Phase I	NCT02687386
	MRX34 (mir-34a mimic)	Only	Primary liver cancer, SCLC, NSCLC, lymphoma, melanoma	Terminated	Phase I	NCT01829971
	TargomiRs	Only	MPM, NSCLC	Completed	Phase I	NCT02369198
	Patisiran	Only	hereditary transthyretin amyloidosis	Completed	FDA approved	[287]
	Cobomarsen (anti-mir155)	Only	CTCL, CLL, ATLL	Completed	Phase I	NCT02580552
		+ Vorinostat	Cutaneous T cell lymphoma/mycosis fungoides	Terminated	Phase II	NCT03713320

## Conclusion

Cancer resistance and recurrence is one the most important pressing concerns in conventional cancer treatments. Scientists are aware that there is a small population of CSCs in different tumors that create resistance to radiotherapy and chemotherapy, which can be attribute to the abilities to self-renew, differentiate and proliferate indefinitely. These cells can exit the cell cycle for a long time and remain in a quiescent state, rendering treatment difficult and allowing the cells to survive conventional therapies [288]. The dormant phenotype can remain in the CSCs for decades, eventually leading to recurrence of the tumor. In fact, one of the main causes of death in cancer has been reported to be the dormancy state of CSCs, which can cause metastasis and recurrence of the disease after several decades [23, 130, 289]. In addition, due to the ability to change the cell cycle checkpoints and the efficient DNA damage repair system thereof, CSCs have high survival capacity, which makes them resistant to chemotherapy drugs such as cisplatin, oxaliplatin, doxorubicin, daunorubicin and methotrexate [7, 16]. Moreover, CSCs can escape apoptosis by mutating and inactivating apoptotic genes, such as *p53*, and the overexpression of anti apoptotic proteins, such as AKT and BCL2. High activity of Notch and hedgehog signaling pathways can also activate anti-apoptotic signaling pathways in CSCs [7, 290, 291].

Acting as one of the main agents of MDR in cancers, the overexpression of ABC transporters such as ABCB1, ABCC1, and ABCG2 have been reported in CSCs that protect cells from cytotoxic agents. Abnormal epigenetic changes and signaling pathways directly or indirectly affect their ABC expression in CSCs [117, 290]. Studies have revealed that ABC transpoters expression is increased by promoter hypomethylation of these genes. This can be seen in the promoter methylation of the ABCG2 transporter regulating the expression in CSCs. Further observations have been made that increasing the activity of the DNMTs by melatonin decreased the expression of this gene in brain CSCs through hypermethylating the ABCG2 promoter [292], while ABCC1 overexpression was also observed to be regulated in the CSCs by activating the Notch pathway, which causes cell resistance [288].

Hence, considering the role of CSCs in therapeutic resistance, recognizing the characteristics of CSCs among other tumor cells and the factors affecting the formation will be beneficial in the design of targeted drugs for cancer. In the present review, recent information about the biology and characteristics of CSCs was explored, including CSCs markers, CSCs niche, the relationship between EMT and CSCs, the factors that create resistance and influencing the generation of them. An integral factor in the formation and maintenance of CSCs in the epigenetic modifications that promote tumorgenesis and metastasis by deregulation of gene expression and vital cell signaling pathways. Numerous studies have demonstrated that the deregulation of DNA methylation, histone modifications, RNA methylations, miRNAs and LncRNAs in stem cells or adult cells will downregulate the expression of tumor-suppressor genes, upregulate the expression of oncogenes, activate the expression of CSCs surface markers and ultimately induce CSCs formation pathways and maintain the properties of them which is achieved by altering cell function and increasing plasticity, self-renewal, differentiation, and survival in the cells. These abnormal epigenetic changes also affect the expression and function of ligands or signaling pathway items in connection with the preservation the CSC characteristics such as Wnt/β-catenin, Notch, Hedgehog, and TGF-β/BMP. Aberrant activities of aforementioned signaling pathways effectuated by increasing the genes expression has been demonstrated to contribute to the maintenance of phenotype and function of CSCs, which increases the population thereof and has a critical function in the initiation, progression and invasion of various types of cancer. By inducing EMT and overexpression of metastasis-related genes therein, deregulation of epigenetic alternation can also increase the ability of metastasis and invasion of CSCs to different tissues, causing disease relapse even after therapy. Therefore, these epigenetic changes provide promising prospects for treatment and have been considered by scientists to generally eliminate CSCs and overcome chemotherapy resistance. In particular, recognizing epigenetic signatures in different genes in CSCs, which are currently considered as clinical biomarkers [293–295], is integral to the prevention and treatment of cancer. In existing research, epigenetic inhibitors have exhibited favorable results in treatment. In addition, several epi-drugs are at various stages of clinical trials and some have been approved by the FDA, such as decitabine, azacitidine, and vorinostat, with the combination of these inhibitors showing synergistic effects and favorable results in treatment. However, further research is required to determine the more specific features of CSCs in the design of targeted treatment strategies, so that treatment is performed directly on CSCs rather than on stem cells similar thence. Because the niche of CSCs is related to the niche of normal stem cells, this has led to a lack of specific treatment in some cases and also involved healthy tissues [296]. Thus, to prevent recurrence and resistance of various cancers and increase the lifespan of the patient, one of the greatest strategies in cancer treatment is to target CSCs directly and specifically.

## Data Availability

The datasets generated and analyzed during the current study are available from the corresponding authors on reasonable request by permission of institute and department chairman’s.

## Abbreviations

ABC: ATP-binding cassette
ABCG2: ATP-binding cassette sub-family G member 2
AML: Acute myeloid leukemia
ALDH1: Adehyde dehydrogenase1
APC: Adenomatous polyposis coli
ASCL1: Achaete-scute family BHLH transcription factor 1
ATLL: Adult T cell leukemia/lymphoma
Aza: 5-Azacitidine
AzadC: 5-Aza-2′-deoxycytidine
BCSCs: Breast cancer stem cells
BMI1: B-Lymphoma Mo-MLV insertion region 1 homolog
BMPs: Bone morphogenetic proteins
CAFs: Cancer-associated fibroblasts
CLL: Chronic lymphocytic leukemia
CMML: Chronic myelomonocytic leukemia
CNS: Central nervous system
CRC: Colorectal cancer
CSC: Cancer stem cells
CSCC: Cervical squamous cell carcinoma
CTCL: Cutaneous T cell lymphoma
DKK1: Dickkopf-related protein 1
Dll: Delta-like
DLK1: Delta‐like 1 homolog
DNMTs: DNA methyltransferases
DNMTis: DNMT inhibitors
DZNep: 3-Deazaneplanocin A
ECM: Extracellular matrix
EGF: Epithelial growth factor
EMA: European Medicines Agency
EMT: Epithelial-to-mesenchymal transition
EpCAM: Epithelial cell adhesion molecule
ERCC1: Excision repair cross-complementation group 1
ESCs: Embryonic stem cells
EZH2: Enhancer of zeste homolog 2
FAD: Flavin adenine dinucleotide
FDA: Food and Drug Administration
FGF: Fibroblast growth factor
GBM: Glioblastoma multiforme
Gli: Glioma-associated oncogene
H3Ac: Acetylated histones H3
H3K27: Histone H3 lysine 27
H3K36: Histone H3 lysine 36
H3K4: Histone H3 lysine 4
H3K79: Histone H3 lysine 79
H3K9: Histone H3 lysine 9
H3K27me3: Trimethylation of H3K27
H4K20: Histone H4 lysine 20
HAT: Histone acetyltransferases
HCC: Hepatocellular carcinoma
HDAC: Histone deacetylase
HDACis: HDAC inhibitors
HES: Hairy/Enhancer of Split
HGF: Hepatocyte growth factor
Hh: Hedgehog
HIFs: Hypoxia-inducible factors
HKMT: Histone lysine methyltransferase
HMT: Histone methyltransferases
HNSCC: Head and neck squamous cell carcinoma
IL-6: Interleukin-6
GSCs: Glioblastoma stem cells
GNRH: Gonadotropin-releasing hormone
KDM: Histone lysine demethylase
lncRNAs: Long noncoding RNAs
LSCs: Leukemic stem cells
m6A: N6-methyladenosine
MDS: Myelodysplastic syndrome
METTL3: Methyltransferase-like 3
miRNAs: Micro-RNAs
MLL: Mixed lineage leukemia
MLL2: Mixed lineage leukemia protein 2
MPM: Malignant pleural mesothelioma
MSCs: Mesenchymal stem cells
ncRNAs: Non coding RNAs
NER: Nucleotide excision repair
NF-κB: Nuclear factor kappa b
NICD: Notch intracellular domain
NSCLC: Non-small cell lung cancer
NSCs: Neural stem cells
OCT4: Octamer-binding transcription factor 4
OSCC: Oral squamous cell carcinoma
PcG: Polycomb-group proteins
PCSCs: Pancreatic CSCs
PTCH1: Patched receptor
RCC: Renal cell carcinoma
PRC2: Polycomb repressive complex 2
SAHA: Suberoylanilide hydroxamic acid
SFRP: Several frizzled-related protein
Shh: Sonic hedgehog ligand
SiRNA: Small interfering RNAs
SCLC: Small cell lung cancer
SMAD Proteins: Sma and Mad proteins
SMO: Smoothened
SNAIL: Snail family zinc finger 1
SOX2: SRY-box 2
STRAP: Serine–threonine kinase receptor-associated protein
TGF-β: Transforming growth factor-β
TGFbR2: TGFb-receptor-2
TICs: Tumor initiating cells
TNBC: Triple-negative breast cancer
TNF: Tumor necrosis factor
TWIST1: Twist-related protein 1
WIF-1: Wnt inhibitory factor 1
WTAP: Wilms tumor 1 associated protein
ZEB1: Zinc finger E-box-binding homeobox 1
